# Mitochondrial oxidative damage reprograms lipid metabolism of renal tubular epithelial cells in the diabetic kidney

**DOI:** 10.1007/s00018-023-05078-y

**Published:** 2024-01-11

**Authors:** Yanjuan Hou, Enxue Tan, Honghong Shi, Xiayu Ren, Xing Wan, Wenjie Wu, Yiliang Chen, Hiumin Niu, Guozhen Zhu, Jing Li, Yafeng Li, Lihua Wang

**Affiliations:** 1 Department of Nephrology, Second Hospital, Shanxi Medical University, No.382, Wuyi Road, Taiyuan, Shanxi 030000 China; 2 Department of Orthopaedics, Second Hospital, Shanxi Medical University, Taiyuan, China; 3 Department of Medicine, Medical College of Wisconsin, Milwaukee, WI USA; 4 Versiti Blood Research Institute, Milwaukee, WI USA; 5 Department of Nephrology, Heping Hospital, Changzhi Medical College, Changzhi, China; 6 Department of Nephrology, Shanxi Province People’s Hospital, Taiyuan, China; 7 Shanxi Provincial Key Laboratory of Kidney Disease, Taiyuan, China

**Keywords:** Diabetic kidney disease, Renal tubular epithelial cells, Mitochondrial oxidative damage, Fatty acid oxidation, MtROS

## Abstract

The functional and structural changes in the proximal tubule play an important role in the occurrence and development of diabetic kidney disease (DKD). Diabetes-induced metabolic changes, including lipid metabolism reprogramming, are reported to lead to changes in the state of tubular epithelial cells (TECs), and among all the disturbances in metabolism, mitochondria serve as central regulators. Mitochondrial dysfunction, accompanied by increased production of mitochondrial reactive oxygen species (mtROS), is considered one of the primary factors causing diabetic tubular injury. Most studies have discussed how altered metabolic flux drives mitochondrial oxidative stress during DKD. In the present study, we focused on targeting mitochondrial damage as an upstream factor in metabolic abnormalities under diabetic conditions in TECs. Using SS31, a tetrapeptide that protects the mitochondrial cristae structure, we demonstrated that mitochondrial oxidative damage contributes to TEC injury and lipid peroxidation caused by lipid accumulation. Mitochondria protected using SS31 significantly reversed the decreased expression of key enzymes and regulators of fatty acid oxidation (FAO), but had no obvious effect on major glucose metabolic rate-limiting enzymes. Mitochondrial oxidative stress facilitated renal Sphingosine-1-phosphate (S1P) deposition and SS31 limited the elevated Acer1, S1pr1 and SPHK1 activity, and the decreased Spns2 expression. These data suggest a role of mitochondrial oxidative damage in unbalanced lipid metabolism, including lipid droplet (LD) formulation, lipid peroxidation, and impaired FAO and sphingolipid homeostasis in DKD. An in vitro study demonstrated that high glucose drove elevated expression of cytosolic phospholipase A2 (cPLA2), which, in turn, was responsible for the altered lipid metabolism, including LD generation and S1P accumulation, in HK-2 cells. A mitochondria-targeted antioxidant inhibited the activation of cPLA2f isoforms. Taken together, these findings identify mechanistic links between mitochondrial oxidative metabolism and reprogrammed lipid metabolism in diabetic TECs, and provide further evidence for the nephroprotective effects of SS31 via influencing metabolic pathways.

**Supplementary Information:**

The online version contains supplementary material available at 10.1007/s00018-023-05078-y.

## Introduction

Diabetic kidney disease (DKD) is one of the major causes of end-stage kidney disease, and is thus a crucial challenge for public health worldwide [1]. In recent years, with the recognition of the "tubulocentric view of DKD", the importance of metabolic changes to tubular epithelial cells (TECs) and mitochondrial damage in the pathogenesis of DKD has received increased attention [2, 3]. Systemic metabolic disorders initiated by hyperglycemia cause TEC metabolic alterations, which might be related to mitochondrial dysfunction and lead to fibrosis progression [4–7]. It has long been postulated that TECs exhibit persistently elevated mitochondrial oxidative stress levels, which occurs even at the early stage of DKD [8]. Recent evidence indicated that an altered pro-oxidant shift can promote metabolic alterations by inducing DNA damage and genomic instability [9, 10]. Mitochondrial reactive oxygen species (mtROS) also act as second messengers to regulate metabolic pathways by directly or indirectly affecting the activity or stability of various metabolic enzymes [11, 12]. However, the influence of mitochondrial oxidative stress and dysfunctional mitochondria in cellular metabolic reprogramming, and the related mechanism in diabetic TECs, is not completely understood.

Mitochondrial fatty acid β-oxidation (FAO) is the preferred source of adenosine triphosphate (ATP) in TECs in the healthy kidney [4]. Reprogrammed lipid metabolism of TECs, including changes in fuel-source preferences, defective FAO, and elevated lipotoxicity, has emerged as an important mechanism of multiple cellular injury, in which interactions with interstitial fibrosis create a vicious circle that promotes the development of DKD [13]. Recent studies have shown that not only the quantity of ectopic lipid deposition (ELD), but also lipid species alterations, induced devastating outcomes in lipotoxicity-associated renal damage [14]. Metabolomic analyses in clinical and animal nephropathies have described sphingolipid accumulation and the intracellular sphingolipid composition of renal cells as important determinants of renal function [15]. Sphingosine-1-phosphate (S1P), as a bioactive sphingolipid metabolite that mediates inter- and intracellular signaling, is implicated in renal injury [16]. Sphingosine kinase 1 (SPHK1) is an important signaling enzyme that catalyzes the substrate-level phosphorylation of sphingosine to generate S1P and is involved in various signaling pathways associated with its serine acetyltransferase activity [16]. Evidence indicates that S1P signaling is associated with acceleration of lipotoxic stress, as well as playing roles in regulating biological processes, such as cell growth, differentiation, migration, and apoptosis, in a wide variety of cells types [17–19]. Importantly, mitochondria play an important role in the generation of metabolites, and the dysregulation of mitochondrial function increases intracellular oxidation associated with the changes of all these lipid metabolic pathways [3, 4, 6, 8]. Therefore, exploring the mechanistic link between mitochondrial oxidative damage and altered lipid metabolism in TECs will increase our understanding of the pathophysiology of DKD.

In recent years, the crucial role of phospholipase A2 (PLA2) in maintaining lipid metabolism homeostasis has received increased attention, especially in metabolic diseases [20]. PLA2 activation results not only in the degradation of membrane phospholipids, but also in the accumulation of unsaturated free fatty acids, which can injure cells [21]. Among the isoforms of PLA2, cytosolic PLA2 (cPLA2, group IV PLA2) has been proposed in numerous pathophysiological processes in the kidney, as evidenced by alterations in renal disorders such as diabetic nephropathy, glomerulonephritis, and ischemic injury [22–24]. cPLA2 mediates the cytotoxicity and apoptosis resulting from tumor necrosis factor in the ischemic kidney during damage to TECs, and inhibition of cPLA2 activity is considered an advantageous strategy to prevent and treat tubulointerstitial inflammation and fibrosis in chronic kidney disease [24, 25]. Moreover, studies in different diseases have also documented that oxidant stress and peroxidation of lipid substrates can enhance cPLA2 activity, and this activation of cPLA2 can be a critical factor in the generation of cellular lipid peroxides and injury [26]. Scavengers of non-specific ROS attenuate downstream pathways of cPLA2 in obesity-related tubule interstitial injury [27]. Although cPLA2 activity is altered in damaged TECs, in which the most relevant phenomenon is oxidative injury, little is known about the role of cPLA2 in controlling lipid homeostasis and the potential impact of mitochondrial oxidative stress in this process during DKD.

In this study, we explored the role of dysfunctional mitochondria and oxidative stress in metabolic alterations of TECs during DKD. We demonstrated that mitochondrial oxidative damage-promoted ELD and lipid peroxidation contributed to diabetic renal tubular injury. We further determined that LD formation and S1P accumulation are involved in TEC injury, which could be attenuated by inhibition of cPLA2 activation. A mitochondria-targeted antioxidant provided critical protection against the activation of cPLA2 isoforms in diabetic TECs. These studies provide proof of concept for therapeutic reprogramming of TEC lipid metabolism by inhibiting mitochondrial oxidative damage for mitochondrial protection in the diabetic kidney.

## Materials and methods

### Experimental animals

Male C57BLKS/J db/db diabetic (*n* = 12) and littermates of non-diabetic heterozygous db/m (*n* = 6) mice aged 8 weeks were purchased from the Model Animal Research Center of Changzhou Cavens (Jiangsu, China). The animals were housed in Shanxi Medical University with a temperature of 21–25 ℃, humidity of 50–60%, a 12 h:12 h light–dark cycle, and free access to a regular diet and pure drinking water. Half of the db/db mice (*n* = 6) were injected intraperitoneally with 3 mg/kg SS-31 (D-Arg-2′6′-dimethylTyr-Lys-Phe-NH2; ChinaPeptides, Shanghai, China) daily for 10 weeks. The dosage (3 mg/kg/day) was based on related studies showing the efficacy of SS31 without adverse effects [28]. The db/m mice and the other half of the db/db mice (*n* = 6) were injected with a 0.1 mol/L sodium citrate solution. The mice were euthanized at 18 weeks old, and serum, urine, and kidneys were harvested for further biochemical and histological analysis. All experimental protocols were conducted according to the Ethics Review Committee for Animal Experimentation of Shanxi Medical University.

### Cell culture and transfection

The human renal proximal tubular cell line (HK-2) was obtained from the ATCC (American Type Culture Collection, Manassas, VA, USA) and maintained in Dulbecco’s modified Eagle’s medium (DMEM) containing 1 g/L glucose supplemented with 10% fetal bovine serum (FBS), 100 U/mL penicillin, and 100 mg/mL streptomycin at 37 °C in a 5% CO_2_ atmosphere. D-glucose, mannitol, palmitic acid (PA), S1P, and triphenyl-phosphonium chloride (Mito-Tempo) were purchased from Sigma (St. Louis, MO, USA). The PLA2 activator (PLAP) was purchased from Santa Cruz Biotechnology (Dallas, TX, USA). HK-2 cells were fasted for 12 h and then stimulated with normal glucose (NG) 5.6 mmol/L, NG plus 24.4 mM mannitol (M), 30 mmol/L glucose (HG), HG plus 100 nM SS31 (HG + SS31), HG plus 25 μM Mito-Tempo (HG + Mtmp) for 72 h. For the PA, S1P, and PLAP intervention groups: HK-2 cells were exposed to HG or not, but treated with 300 μM PA (PA) or 0.5, 1, 2 μM S1P (S1P), or 1 μM PLAP (PLAP) for 24 h. The expression of *SPHK1* and *CPLA2* was knocked down by the transfection of short hairpin RNA (shRNA) plasmids or a control shRNA plasmid (Genechem, Shanghai, China) into HK-2 cells using an FuGENE-HD transfection reagent (Fugent LLC, Middleton, WI, USA) in accordance with the manufacturer's instructions. The inhibition efficiencies of the shRNAs were confirmed using western blotting.

### Metabolic parameters

Blood glucose (BG), kidney weight (KW), body weight (BW), urine volume, albumin concentrations, and blood lipids were measured in the mice at 18 weeks old. The level of urinary albumin, serum creatinine (Scr), serum total cholesterol (TC), and serum triglycerides (TG) waas determined using reagent kits (BioSino Bio-technology and Science Inc., Beijing, China) in accordance with the manufacturer’s instructions.

### ROS detection

Mitochondrial superoxide generation was detected using the specific mitochondria-targeted superoxide fluorescent probe, MitoSOX Red (Thermo Fisher Scientific, Waltham, MA, USA). Single cell suspension of tubular cells from renal cortex tissues was prepared as described previously [29]. Briefly, kidneys were minced and incubated with collagenase I (Thermo Fisher Scientific). Digested kidney cells were filtered through the 100 μm, 70 μm, and 40 μm meshes. Cells were centrifuged and follow by incubated in Red Blood Cell (RBC) lysis buffer (Boster Biological Technology co. Ltd, Wuhan, China) to remove the erythrocytes. Then, the cells were resuspended in RPMI 1640 medium (Thermo Fisher Scientific) for ROS detection. HK-2 cells were seeded into 36 mm cell culture dishes and subjected to various treatments. The single cell suspension of the kidney tissues or HK-2 cells was incubated with a 5 μM MitoSOX working solution at 37 °C for 30 min before flow cytometry. The measurement of mtROS performed using flow cytometry (BD Immunocytometry Systems, Franklin Lakes, NJ, USA), and the analysis was performed using FlowJo software (FlowJo LLC, Ashland, OR, USA). The median fluorescence intensity was used to estimate the average amount of superoxide production.

### Measurement of lipid accumulation

The triglyceride content in renal cortical tissues or HK-2 cells was measured using quantification kits (Jiancheng Bioengineering, Nanjing, China) according to instructions of the manufacturer. Frozen kidney tissue Sects. (8 µm-thick) were fixed in 4% paraformaldehyde for 30 min and then stained with 0.3% Oil Red O (Sigma) solution for 15 min at room temperature. After rinsing with 60% isopropanol and washing three times with PBS, the cells were counterstained with hematoxylin for 5 min and examined under light microscopy. Lipid droplets in kidney tissues were also evaluated using electron microscopy. Kidney tissue was fixed in 2.5% glutaraldehyde and 1% osmium tetroxide, washed with PBS, and dehydrated via a series of graded ethyl alcohol solutions (50%, 70%, 90%, and 100%). After exchange through acetone, the samples were embedded in Quetol 812 mixture (Nissin, Tokyo, Japan). The samples were detected by electron microscopy at the Kingmed medical Test center (Taiyuan, China). A transmission electron microscope (Hitachi, Tokyo, Japan) was used to examine and photograph the sections. Lipid droplets in HK-2 cells were stained with BODIPY 493/503 reagent (Thermo Fisher). HK-2 cells were incubated with the dye (20 μL in 1 mL of PBS) for 45 min at 37 °C, and counterstained with 4′,6-diamidino-2-phenylindole (DAPI) (1:20,000; Invitrogen Corporation, Waltham, MA, USA) for 5 min at room temperature. Cells were then imaged using a confocal microscope (Olympus FV 1000 Viewer; Olympus, Tokyo, Japan).

### Enzyme-linked immunosorbent assay (ELISA)

The urinary neutrophil gelatinase-associated lipocalin (NGAL), N-acetyl-alpha-glucosaminidase (NAG), and kidney injury molecule 1 (KIM1) were quantified using a commercial Quantikine Enzyme-Linked ImmunoSorbent Assay (ELISA) kit (R&D systems Minneapolis, MN, USA), in accordance with the manufacturer’s description.

### Renal histopathology and Immunofluorescence staining

Paraffin-embedded kidney Sectons (4 μm-thick) were stained with periodic acid-Schiff (PAS) and examined by light microscopy. Staining was quantified using ImageJ software (National Institutes of Health (NIH), Bethesda, MD, USA). For renal tissue immunofluorescence staining, frozen kidney Sections (8 μm-thick) were prepared, fixed in acetone for 5 min at room temperature, and incubated with primary antibodies overnight at 4 °C. For cell immunofluorescence staining, HK-2 cells were plated on cover slips, fixed with 4% formaldehyde for 15 min at 4 °C, and blocked with 5% bovine serum albumin (BSA) for 30 min. The cells were incubated in 0.1% Triton X-100 for 20 min at room temperature to permeabilize the cell membrane, and then incubated with primary antibodies overnight at 4 °C. Thereafter, the cells were exposed to secondary antibodies for 1 h at 37 °C. Finally, the cell nuclei were stained using DAPI and immediately visualized under a fluorescence microscope (Olympus BX63). The primary antibodies used in this study are listed in Supplementary Table 1.

### Western blotting analysis

The total proteins were extracted from mouse renal cortex tissue and HK-2 cells using Radioimmunoprecipitation assay (RIPA) lysis buffer. The protein concentration was determined using a bicinchoninic acid (BCA) protein assay kit (Beyotime, Jiangsu, China). Western blotting analysis was performed as described previously [28]. The primary antibodies used in this study are listed in Supplementary Table 1. The secondary antibody used was either anti-mouse IgG or anti-rabbit IgG (1:10,000 dilution, Santa Cruz Biotechnology, Santa Cruz, CA, USA) and the blots were scanned using an Odyssey Fc System (LI-COR, Lincoln, NE, USA). The intensity of the immunoreactive protein bands was quantified and analyzed using Image J software.

### RNA Extraction and quantitative real-time reverse transcription PCR (qRT-PCR)

The total RNA from cultured HK-2 cells or renal cortex tissue was prepared using the TRIzol reagent (Invitrogen), followed by reverse transcription to cDNA using a High-Capacity cDNA Reverse Transcription Kit (Takara Bio Inc., Dalian, China) according to the manufacturer’s instructions. The quantitative real-time PCR (qPCR) analysis step of the qRT-PCR protocol was performed using SYBR Premix ExTaq (Takara Bio Inc.) with the cDNA as the template, as previously described [28]. 18 s rRNA was used as the normalization control. The sequences of the primers used in this study are listed in Supplementary Table 2.

### Measurement of the oxygen consumption rate (OCR)

Primary mouse tubular epithelial cells from renal cortex tissues were extracted first, as described previously [29]. Kidneys were dissected, placed in ice-cold Dulbecco's PBS (DPBS) and minced into pieces. Fragments were transferred to a 50 mL tube containing 10 mL of Roswell Park Memorial Institute (RPMI) 1640 medium with 100 μl of Collagenase I and digested for 30 min at 37 °C in a 160/170 rpm shaker. Thereafter, 100 μl of FBS (Thermo Fisher Scientific) was added to stop the Collagenase I reaction. Cells were further sieved through a 100 μm nylon mesh, followed by 70 μm and 40 μm nylon meshes. Cells were centrifuged for 10 min at 3000 g. The pellet was resuspended in 1 mL of sterile RBC lysis buffer and incubated for 2–3 min on ice. DPBS was added, followed by centrifugation for 10 min at 3000 g. Cells were cultured in RPMI 1640 medium supplemented with 10% FBS, 20 ng/mL epidermal growth factor (EGF) (PeproTech, Inc., Cranbury, NJ, USA), 1% Insulin-Transferrin-Selenium (Procell Life Science &Technology Co., Ltd., Wuhan, China) and 1% penicillin–streptomycin (Beijing Solarbio Science & Technology Co., Ltd., Beijing, China) in a humidified atmosphere containing 5% CO_2_ at 37 °C. The medium was changed every two days. On the fourth day, mouse primary renal TECs were seeded into a specialized XF24 cell culture microplate (Seahorse Bioscience, Billerica, MA, USA) at a density of 50,000–80,000 cells/well for OCR detection. The OCR was assessed at baseline and after the addition of palmitate-conjugated BSA (180 µM), followed by the addiction of the carnitine palmitoyltransferase-1 (CPT1) inhibitor etomoxir (4.0 µM), and the ATP synthase inhibitor oligomycin (1.5 µM). After cell lysis in RIPA buffer, a Bradford protein assay was performed. OCR values were normalized to the protein content of each well. We tested the PA-dependent OCR as previously described [30].

### Measurement of metabolites in kidney tissue using liquid chromatography–mass spectrometry (LC–MS)

Kidney tissues were thawed on ice, and the metabolites were extracted using 50% methanol buffer with low-temperature ultrasound. The proteins were then precipitated for 1 h at − 20 °C and centrifuged at 13,000×*g* at 4 °C for 15 min to obtain the supernatant. All samples were acquired by the LC–MS system following the instrument's instructions. First, all chromatographic separations were performed using a Thermo Scientific UltiMate 3000 high performance liquid chromatography apparatus. An ACQUITY UPLC BEH C18 column (100 mm × 2.1 mm, 1.8 μm, Waters, Wilmslow, UK) was used for the reversed phase separation. The column oven was maintained at 35 °C. The flow rate was 0.4 mL/min and the mobile phase consisted of solvent A (water, 0.1% formic acid) and solvent B (Acetonitrile, 0.1% formic acid). Gradient elution conditions were set as follows: 0–0.5 min, 5% B; 0.5–7 min, 5% to 100% B; 7–8 min, 100% B; 8–8.1 min, 100% to 5% B; 8.1–10 min, 5% B. The injection volume for each sample was 4 μL. A high-resolution tandem mass spectrometer Q-Exactive (Thermo Scientific) was used to detect metabolites eluted form the column. The Q-Exactive was operated in both positive and negative ion modes. Precursor spectra (70–1050 m/z) were collected at 70,000 resolution to hit an AGC target of 3e6. The maximum inject time was set to 100 ms. A top 3 configuration to acquire data was set in DDA mode. Fragment spectra were collected at 17,500 resolution to hit an AGC target of 1e5 with a maximum inject time of 80 ms. To evaluate the stability of the LC–MS during the whole acquisition, a quality control sample (pool of all samples) was acquired after every 10 samples. The original multiple reaction monitoring data from the energy metabolites were extracted and the peak area of each metabolite was obtained.

### Quantification of S1P level and SPHK1 activity in HK-2 cells

Cell lysate was prepared from renal cortex sample and HK-2 cells on ice using lysis buffer according to the standard procedure [31]. The lysates were centrifuged for 10 min (6000×*g*) and the resulting supernatant was used to quantify sphingosine kinase1 activity using a Sphingosine Kinase1 Fluorometric Assay Kit (Biovision, Milpitas, CA, USA) according to the manufacturer’s instructions. Levels of S1P in the cell lysate of HK-2 cells were quantified using a competitive ELISA test according to the manufacturer instructions (Echelon Biosciences, Salt Lake City, UT, USA).

### Cytotoxicity assay

HK-2 cell cytotoxicity assessment against S1P was performed using a Cell Counting Kit-8 (CCK-8; Sigma). Briefly, after treatment, CCK-8 solution (10 μL) was added into the culture medium (100 μL) in the required wells at 37 °C for 2 h. Absorbance in the wells was analyzed at 450 nm (OD450) using a microplate reader (SpectraMax i3x, Molecular Devices, San Jose, CA, USA).

### Statistical Analysis

Data are expressed as the mean ± SD. The statistical analyses were carried out using GraphPad Prism software (Version 8.0, GraphPad Inc., La Jolla, CA, USA). Differences between the groups were analyzed for statistical significance using one-way analysis of variance (ANOVA), followed by a post-hoc test using the Tukey–Kramer method. Pearson’s correlations were calculated using GraphPad software. All experiments were performed at least three times. A threshold *P*-value < 0.05 was considered significant.

## Results

### Mitochondrial oxidative stress contributes to ELD-induced renal tubular injury in db/db Mice

**Fig. 1:**
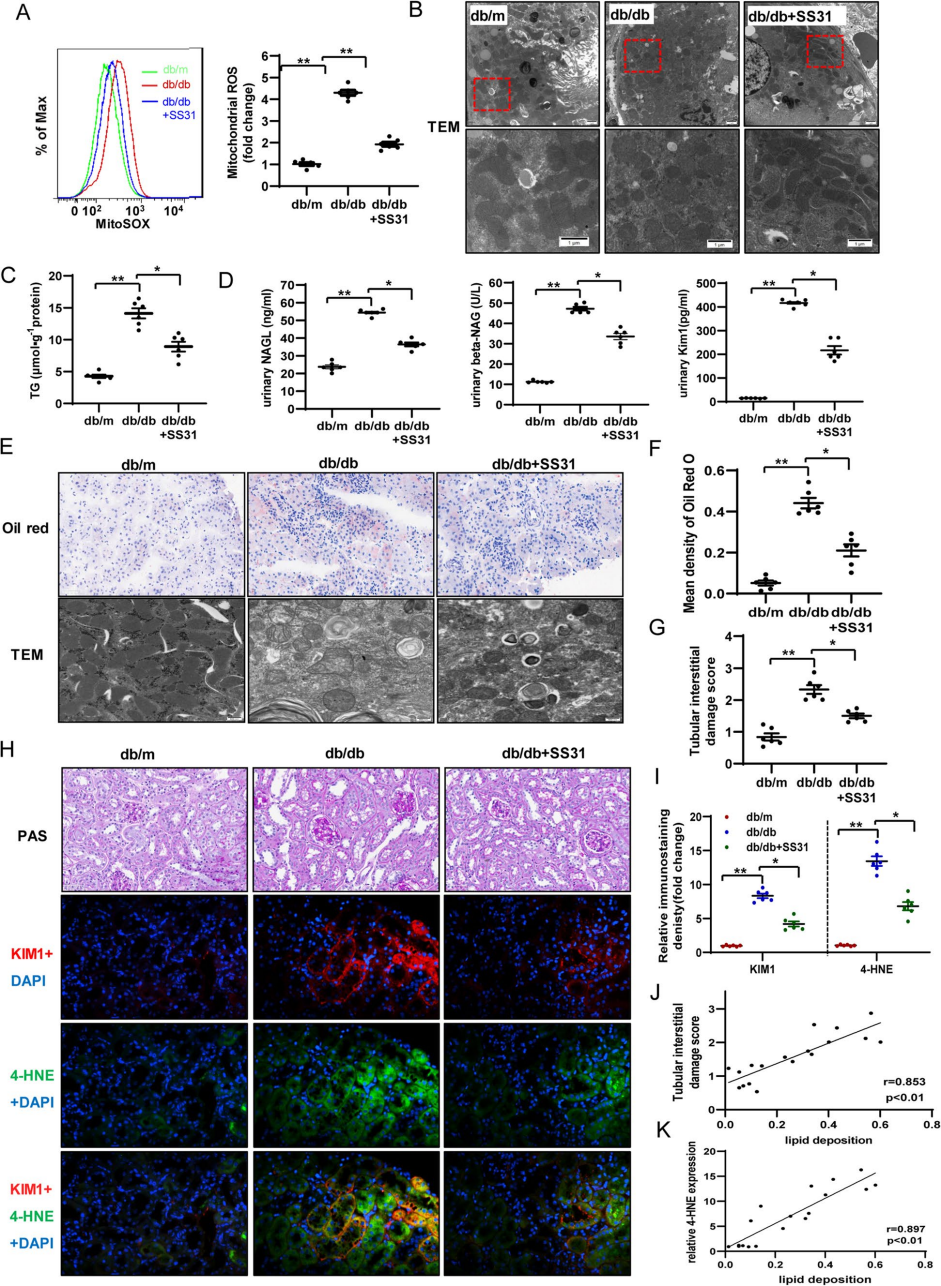
Mitochondrial oxidative stress contributes to ELD-induced renal tubular injury in db/db Mice.** A** Levels of mitochondrial ROS in experimental animals were detected by FACS using the MitoSOX reagent. **B** Representative image of electron microscopy showing changes to mitochondria in kidney cells. **C** Changes in triglyceride levels in the three groups of mice. **D** Changes in renal tubular injury marker levels (NGAL, beta-NAG, Kim- l) in the three groups of mice. **E** Representative images of lipid droplets stained with oil red and electron microscopy images of renal cortex tissue. **F** Quantification of lipid droplets stained with oil red. **G** Quantitative analysis of Tubular interstitial damage scores in each group.** H**, **I** Representative images and quantification for PAS staining, immunofluorescence co-staining of KIM1 and 4-HNE in the three groups of mice. **J**, **K** Correlation of lipid deposition with tubular interstitial damage (**J**) (*P* < 0.01, *R* = 0.853) and 4-HNE (**K** (*P* < 0.01, *R* = 0. 897). db/m: normal male mice; db/db: diabetic mice; db/db + SS31: db/db mice with SS31 treatment; Data are expressed as means ± SD (*n* = 6). **P < 0.01 versus the db/m group; *P < 0.05, compared with the db/db group using ANOVA

To better characterize mitochondrial oxidative damage in TECs, we treated db/m and db/db mice (8 weeks old) with SS31 (a peptide that scavenges mtROS and protects the mitochondrial cristae structure) for 10 weeks. SS31 targets the renal cortex, which is rich in mitochondria, and effectively eliminated renal cortical mtROS and improved mitochondrial damage in diabetic mice, as shown by flow cytometry using MitoSOX (Fig. 1A) and transmission electron microscopy of the renal cortex (Fig. 1B). In our previous work, we showed that SS31 treatment not only restored renal function and morphological changes in DKD, but also decreased lipid accumulation in HG-induced HK-2 cells [28]. In the present study, we found that SS31 suppressed ELD in the renal cortex of db/db mice. This effect was observed using direct measurement of triglyceride in renal cortex tissues (Fig. 1C), Oil red O staining for LDs, and transmission electron microscopy (Fig. 1E, F). The content of LDs located in the proximal tubule compartment was significantly decreased by SS31 treatment (Fig. 1E). However, we did not observe any effects from SS31 on serum lipids, body weight, and blood glucose levels in the db/db mice (Table 1). Therefore, the effect of SS31 on decreased lipid deposition in kidneys was not caused by altered serum lipid levels. These results indicated that mitochondrial dysfunction and mtROS contribute to ELD in kidneys in DKD.

**Table 1:** Change of basic parameters in each group

Group	FBG (mmol L^−1^)	BP (mmHg)	BW (g)	Scr (μmol L^−1^)	TC(mM)	TG(mM)	UAE (μg 24 h^−1^)
db/m	5.12 ± 0.52	121.28 ± 14.23	26.21 ± 0.98	21.49 ± 2.45	2.12 ± 1.25	0.97 ± 0.02	5.41 ± 1.22
db/db	20.85 ± 4.51**	117.98 ± 13.57	35.41 ± 1.03**	48.41 ± 8.01**	5.91 ± 2.01**	2.62 ± 0.82**	261.65 ± 12.23**
db/db + SS31	19.23 ± 2.43	123.52 ± 10.89	32.53 ± 0.84	32.42 ± 2.54*	5.61 ± 3.41	2.32 ± 0.31	161.39 ± 26.43*

Notes: Date are presented as the mean ± SEM (*n* = 6)

*FBG* fasting blood glucose, *BP* blood pressure, *BW* body weight, *Scr* serum creatinine, *TC* total cholesterol, *TG* triglyceride, *UAE* urine albumin excretion

^**^P < 0.05 versus db/m; *P < 0.05 versus db/db

High lipid (triglyceride and long-chain fatty acid) levels in TECs alone are not sufficient to induce cellular injury [4]. ELD-induced lipid peroxidation is more closely related to the renal tubular injury, which interacts with the interstitial fibrosis that promotes disease progression in diabetes [4, 32]. Thus, we further investigated the effect of SS31 treatment on renal tubular injury. We evaluated the urinary NGAL (ng/24 h), urinary NAG (U/L), and urinary KIM1 (pg/mL), comprising three markers of tubular injury, and showed that all their levels increased in the db/db mice, but were significantly decreased in the db/db + SS31 mice (Fig. 1D). Likewise, the levels of KIM1 in kidneys tissues were significantly suppressed in db/db + SS31 mice *vs*. db/db mice (Fig. 1H, I). Immunofluorescent staining and quantitative data showed that KIM1 levels increased in the areas with high levels of 4-hydroxynonenal (4-HNE), a marker of oxidative stress-induced lipid peroxidation and cytotoxicity (Fig. 1H, I). Positive correlations were observed between tubular interstitial damage and lipid deposition (*r* = 0.853) (Fig. 1G, H, J) and between lipid deposition and 4-HNE levels (*r* = 0.837) (Fig. 1H, K) in the kidney tissues of db/db mice. However, treatment with SS31 could decrease KIM1 levels and reduce lipid peroxidation in renal tubules. These results suggested that mitochondrial dysfunction and mtROS in diabetic TECs are involved in ELD-induced renal tubular injury.

### Mitochondrial antioxidants restored the alterations in lipid metabolic pathways in the kidney of db/db Mice

**Fig.2:**
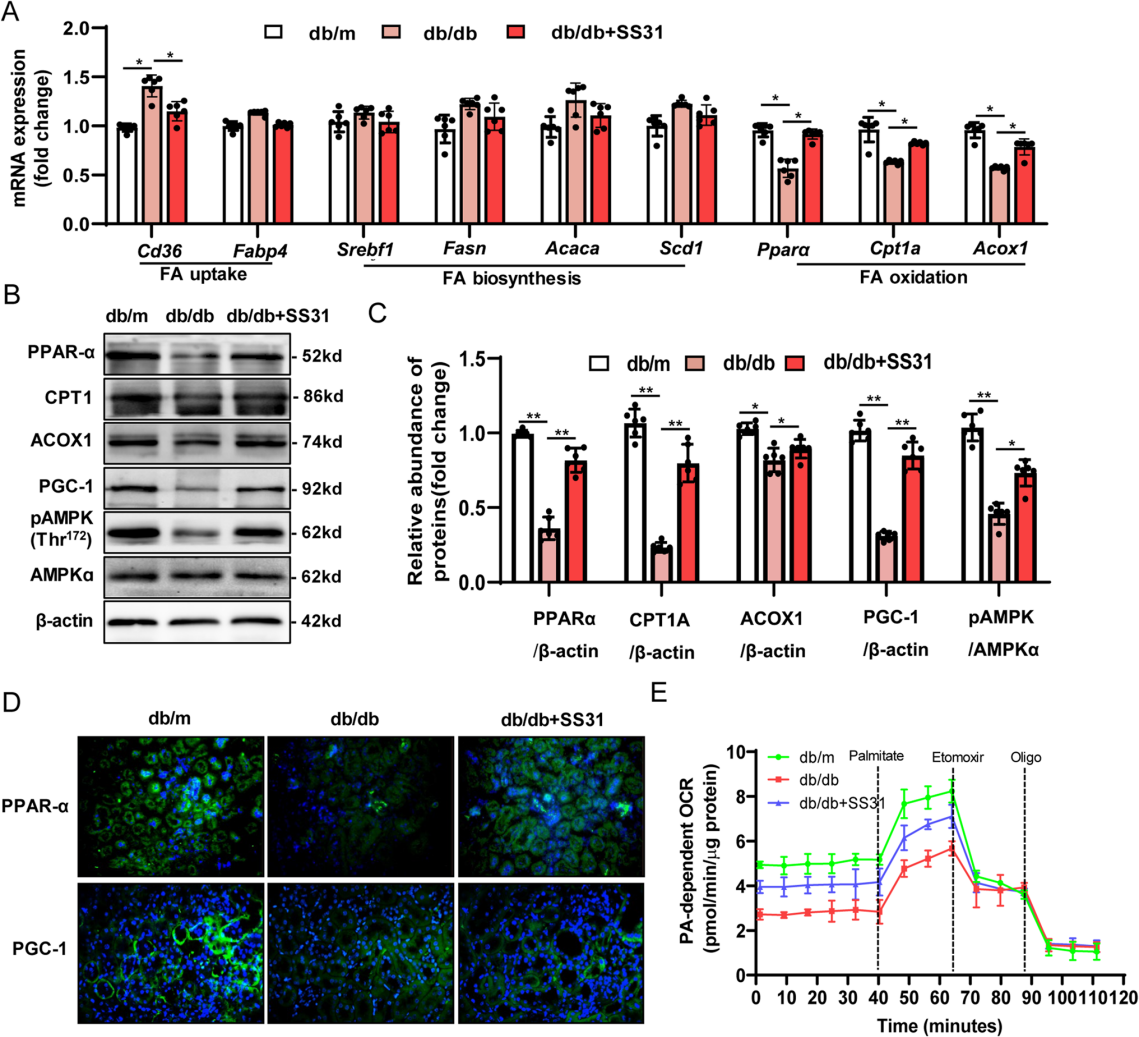
Mitochondrial antioxidants restored the alterations in lipid metabolic pathways in the kidneys of db/db Mice. **A** Renal mRNA levels of Cd36, Fabp4, Srebf1, Fasn, Acaca, Scd1, Pparα, Cpt1a, and Acox1 were detected using qRT-PCR. **B** Representative western blots for PPAR-α, CPT1, ACOX1, PGC-1, pAMPK and AMPKα. **C** Semiquantitative analysis of the PPAR-α, CPT1, ACOX1, PGC-1, pAMPK and AMPKα from the Western blotting data. **D** Immunofluorescence staining of kidney sections with PPAR-α and PGC-1. **E** FAO levels of primary TECs cells were determined by evaluating the PA-dependent oxygen consumption rate (OCR). db/m: normal male mice; db/db: diabetic mice; db/db + SS31: db/db mice with SS31 treatment; Data are expressed as means ± SD (*n* = 6). **P < 0.01 versus the db/m group; *P < 0.05, compared with the db/db group using ANOVA

The occurrence of ELD in renal tubules is mainly related to the imbalance of lipid metabolism, associated with reprogramed enzyme systems in fatty acid uptake, oxidation, intracellular triglyceride synthesis, and lipolysis [33]. To address the contribution of mitochondrial oxidative damage to lipid metabolic pathways in DKD kidneys, we first measured the expression of key genes involved in FA synthesis and uptake in the cortex of db/db mice and compared them to the normal db/m controls. The DKD group had slightly increased FA transporter *Cd36* and *Fabp4* (fatty acid binding protein 4) mRNA levels. The mRNA levels of most of the lipogenic genes that we studied, including *Srebf1* (encoding sterol regulatory element-binding protein 1), *Fasn* (encoding fatty acid synthase), *Acaca* (encoding acetyl CoA carboxylase), and *Scd1* (encoding stearoyl-CoA desaturase) were slightly increased in renal cortex tissues of db/db mice (Fig. 2A). Previous reports in Type 1 diabetic mice and patients with advanced DKD suggested an obvious increase in FA uptake and lipogenesis [34]. Our results showed that important genes in the FA uptake and lipogenic pathway were not significantly increased in 18-week-old db/db mice, which was consistent with other studies [28]. Lipid accumulation could be caused by dysregulation of FAO in the mitochondria, which is a key metabolic phenotype of damaged TECs. We observed a significant suppression in the mRNA and protein levels of the transcriptional master regulator of FAO, peroxisome proliferator activated receptor alpha (PPARα) and its coactivator PPARG coactivator 1 alpha (PGC-1α) in db/db animals, while their expression levels were upregulated by SS31 treatment (Fig. 2A–C). Phosphorylation and activation of AMP-activated protein kinase (AMPK) is also an important regulatory mechanism for fatty acid mitochondrial trafficking and lipid homeostasis [33]. Western blotting analysis showed a significant reduction (about 50%) in phospho-AMPK, an indicator of AMPK activity, in db/db animals; however, the phospho-AMPK level was significantly elevated in SS31-treated animals (Fig. 2A–C). Supporting this finding, genes encoding key steps in β-oxidation, such as *Cpt1a* (encoding carnitine palmitoyltransferase 1A), and peroxisomal oxidation, such as *Acox1* (encoding acyl-coA oxidase 1), returned to nearly 90% normal levels after scavenging mtROS using SS31 (Fig. 2A–C). The microscopic observation of immunofluorescence labeling of PPARα and PGC-1α in the tubular area were consistent with the protein and mRNA results (Fig. 2D). We then assessed the FAO level of primary TECs isolated from mouse kidneys using the FA-driven oxygen consumption rate (PA-dependent OCR). We found that basal and maximum OCRs were markedly higher when we added palmitate to TECs, indicating that TECs efficiently metabolize palmitate. The increase in PA-dependent OCR was markedly reduced by the CPT1 inhibitor etomoxir and blocked by the ATP synthase inhibitor oligomycin-induced blockage of OXPHOS, confirming its specificity. PA-dependent oxygen consumption was higher in primary TECs isolated from db/m control mice. Cells isolated from db/db mice had a lower baseline oxygen consumption level and showed a reduction in PA-dependent OCR, indicating inhibited FAO activity. SS31 treatment was consistently associated with higher PA-dependent oxygen consumption, which was reduced in diabetic db/db mice *vs*. control db/m mice (Fig. 2E), which correlated with increased FAO. Taken together, these results showed that mitochondrial dysfunction-elicited oxidative stress reprograms lipid metabolic pathways by inhibiting FAO activity in the diabetic kidney.

Reprograming of glucose and lipid metabolism in kidney injury is essential to preserve the integrity of kidney mitochondria, thereby preventing massive collateral damage, including chronic inflammation [35]. Previous reports in cultured hepatocytes and other cell types have suggested that ROS stimulates major glucose metabolic rate-limiting enzymes [36]. However, our results suggested that important rate-limiting enzymes of glycolysis (hexokinase 1 (HK1), phosphofructokinase 1 (PFK1), and pyruvate kinase M1/2 (PKM)) were not changed at the mRNA (Fig. Supplementary Fig. 1A) and protein levels (Fig. Supplementary Fig. 1B) by SS31 treatment.

### Mitochondrial oxidative stress alters S1P metabolism and signaling in the kidney of db/db Mice

**Fig. 3:**
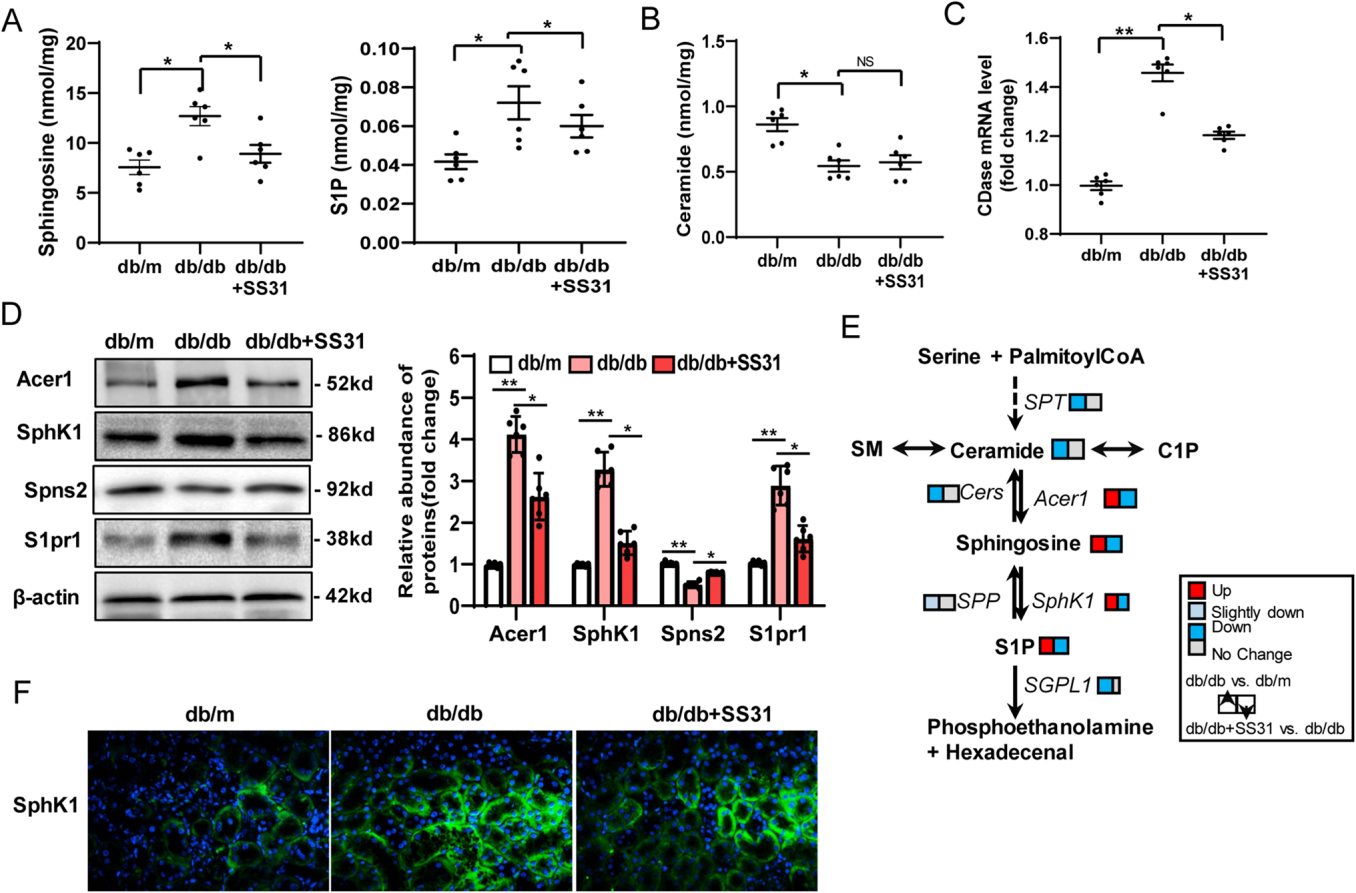
Mitochondrial oxidative stress alters S1P metabolism and signaling in the kidneys of db/db Mice. **A**, **B**: Levels of Sphingosine, S1P, and ceramide (**B**) were determined using LC/MS. **C** Renal mRNA levels of Acer1 were detected using qRT-PCRPCR. **D** Representative western blotting assay and quantitation of the levels of Acer1, SPHK1, Spns2, and S1pr1 in kidney tissues. **E** Schematic representation of the active regulation of S1P metabolism and signaling by prolonged oxidative stress in the diabetic kidney.** F** Representative images of immunofluorescence staining of SPHK1. db/m: normal male mice; db/db: diabetic mice; db/db + SS31: db/db mice with SS31 treatment; Data are expressed as means ± SD (*n* = 6). **P < 0.01 versus the db/m group; *P < 0.05, compared with the db/db group using ANOVA

To gain an overview of the effects of mitochondrial oxidative stress on the metabolic phenotype of diabetic kidneys, we performed a LC/MS analysis using renal cortex tissue. Changes in metabolites between db/db mice that were treated or not treated with SS31 were mainly involved in amino acid metabolism and lipid metabolism (Supplementary Table 3). Unexpectedly, our enrichment analysis did not find significant changes in the fatty acid metabolic pathway. However, sphingosine metabolism was identified as one of the main changes in metabolites between db/db group with or without SS31 treatment (Supplementary Fig. 2A–C). We next quantified the contents of major bioactive sphingolipid metabolites, ceramide, sphingosine, and S1P, in the kidney cortex. We found that sphingosine and S1P markedly accumulated in the kidneys from db/db mice, but not in the control db/m mice kidneys, which was inhibited by SS31 treatment (Fig. 3A). Furthermore, lower amounts of ceramide were also observed in db/db mice compared with those in the control mice; however, ceramide levels were not altered by SS31 (Fig. 3B). These results suggested that mitochondrial oxidative stress might be associated with altered S1P metabolism.

Specific alterations in key and rate-limiting enzymes of sphingolipid metabolism were also measured to verify which mechanisms might be related to the effects of SS31. Notably the mRNA levels of *Acer1* (encoding alkaline ceramidase 1) were significantly increased and the mRNA levels of *Spt* (encoding serine palmitoyltransferase), *Cers2* (encoding ceramide synthase 2 and regulated by S1P [37]), and *Sgpl1* (encoding sphingosine-1-phosphate lyase 1) were significantly decreased in the db/db mice (Fig. Supplementary Fig. 3A). Among these altered metabolic enzymes, only the change in Acer1 could be reversed by SS31 administration (Fig. 3C, D). SPHK1 and signal peptide peptidase (SPP) are involved in S1P remodeling; therefore, we further determined the phosphorylation changes of these two proteins. We found that phosphorylation-mediated activation of SPHK1 markedly increased in the kidney of the db/db mice and was significantly preserved by SS31 treatment, but not for SPP (Fig. 3D, F). These data strongly indicated that mitochondrial oxidative stress plays a role in S1P accumulation in diabetic renal tissue by affecting SPHK1 activation. Given that S1P can be transferred to cytoplasmic organelles that regulate biological processes as second messengers, we then identified the levels of main receptors and transporters of S1P. The results showed that *Spns2* (encoding sphingolipid transporter 2) expression was markedly decreased in db/db mice, and *S1pr1*(encoding sphingosine-1-phosphate receptor 1) expression was increased, both of which could be reversed by SS31 intervention (Fig. 3C). This finding suggested that S1P metabolism and signaling are actively regulated by prolonged oxidative stress in the diabetic kidney (Fig. 3E), a finding that awaits mechanistic investigation.

### S1P promotes lipotoxicity and pro-fibrotic responses in HG-induced HK-2 cells

**Fig. 4:**
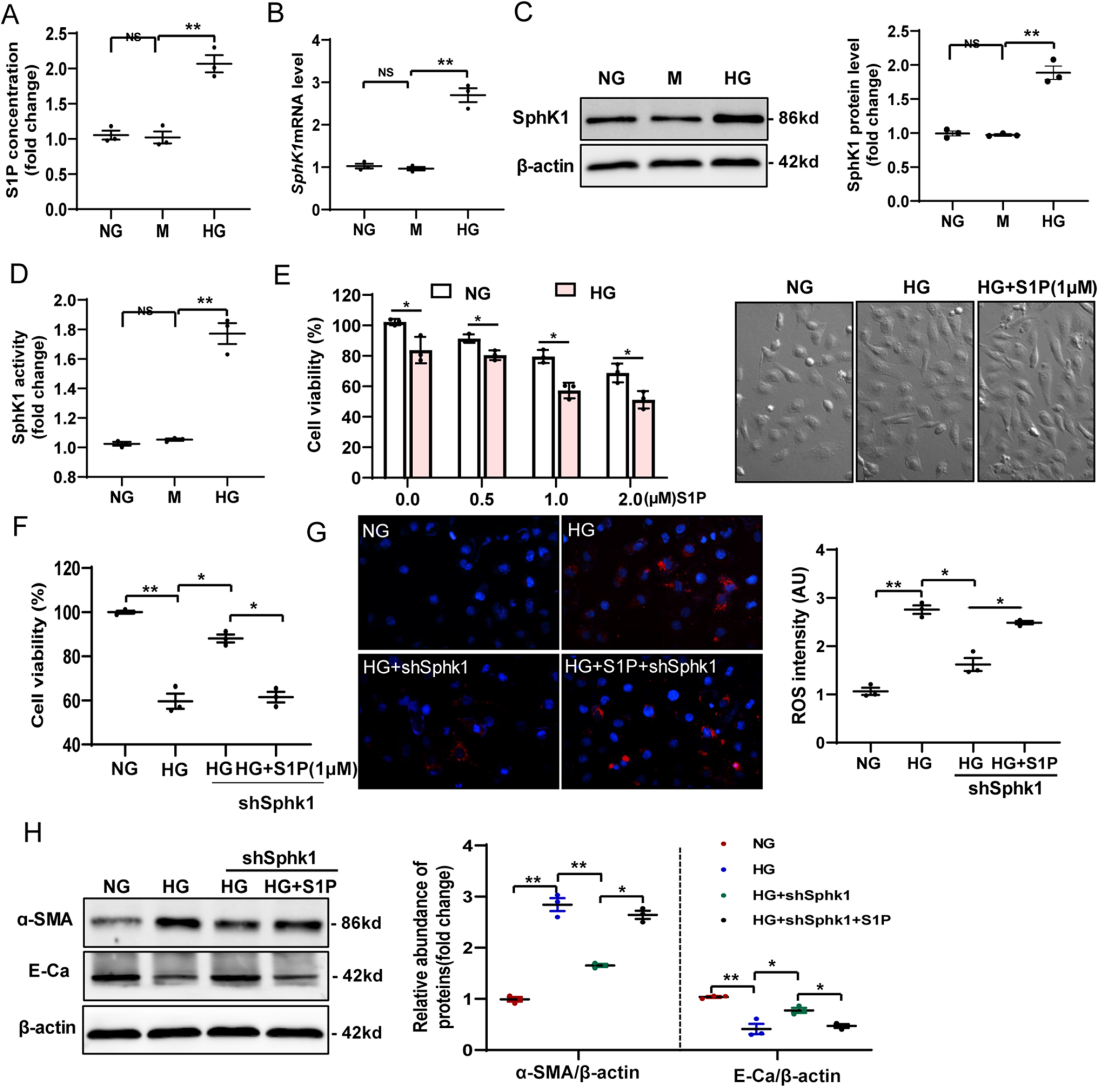
S1P promotes lipotoxicity and pro-fibrotic responses in HG-induced HK-2 cells.** A** Levels of S1P were determined using ELISA. **B** qRT-PCR analysis of SPHK1 mRNA expression in HK-2 cells subjected to HG exposure. **C** Representative western blotting assay and quantitation of the level of SPHK1 in HK-2 cells. **D** SPHK1 activity in HK-2 cells.** E** Cell viability of HG-induced HK-2 cells treated with exogenous S1P. **F** Morphological changes of HK-2 cells were analyzed under an inverted microscope. **G** Mitochondrial ROS generation was assessed using the fluorescence probe MitoSOX Red. **H** Representative western blotting assay and quantitation of the level of ɑ-SMA and E-Cadherin. NG: 5.6 mM D-glucose; M: NG + 24.4 mM mannitol; HG: 30 mM D-glucose; HG + S1P: HG + 1 µM S1P; HG + shSphk1: HG + Sphk1shRNA plasmid, HG + S1P + shSphk1: HG + S1P + Sphk1shRNA plasmid. Data are expressed as the mean ± SD of three independent experiments. **P < 0.01 versus the NG group; #*P* < 0.05, compared with the HG group using ANOVA

To determine whether the increased S1P accumulation in TECs was critical for cellular damage, we first tested the effect of high ambient glucose on S1P levels and SPHK1 activation in the human TEC line, HK-2. Exposure of cells to 30 mM D-glucose for 72 h, but not to an osmotic control, resulted a ∼2.3-fold increased level of S1P production (Fig. 4A). At the same time, SPHK1 mRNA (Fig. 4B) and protein (Fig. 4C) expression in cell lysates increased by ∼2.9-fold and twofold, respectively, in high glucose (HG)-treated HK-2 cells. Along with the increased expression of SPHK1, increased SPHK1 enzymatic activities were detected in HG-stimulated cells compared with those control cells (Fig. 4D). These findings suggested that the SPHK1-S1P pathway was activated in TECs by HG. We next directly evaluated cytotoxicity in HG-induced HK-2 cells treated with exogenous S1P. Cell viability (Fig. 4E) assessment indicated that S1P supplementation for 24 h aggravated HG-induced cell death. To further demonstrate that S1P accumulation promotes lipotoxicity-mediated cellular damage in HK-2 cells under HG conditions, an *SPHK1* shRNA plasmid was transfected into HK-2 cells to inhibit *SPHK1* expression (Fig. Supplementary Fig. 4A, B), followed by incubation with HG for 72 h. The measurements confirmed that knockdown of *SPHK1* suppressed HG-induced cell death (Fig. 4F). Additionally, the MitoSOX assay showed that knockdown of *SPHK1* also suppressed HG-induced ROS production (Fig. 4G). However, these changes induced by *SPHK1* shRNA were attenuated by exogenous S1P treatment.

Lipotoxicity is closely related to pro-fibrotic phenotypic changes in TECs [38]. Therefore, we next determined the levels of smooth muscle actin alpha (α-SMA) and E-cadherin (CDH1), which are markers of epithelial-to-mesenchymal transition, in HK-2 cells treated as above. Figure 4H shows that the levels of α-SMA induced by HG were decreased by shRNA-mediated inhibition of *SPHK1* in HK-2 cells compared with that in the normal glucose (NG) control. Consistently, the addition of exogenous S1P attenuated the decrease in α-SMA levels in response to *SPHK1* shRNA. In contrast, there was a significant increase in CDH1 levels when *SPHK1* expression was abolished by shRNA in HG-stimulated HK-2 cells. Attenuation of the shRNA-induced increase in CDH1 levels was observed when exogenous S1P was administered. These findings indicated the lipotoxic effects of S1P accumulation in TECs under high ambient glucose.

### Elevated cPLA_2_expression is required for LD formulation and S1P accumulation in HG-induced HK-2 cells

**Fig. 5:**
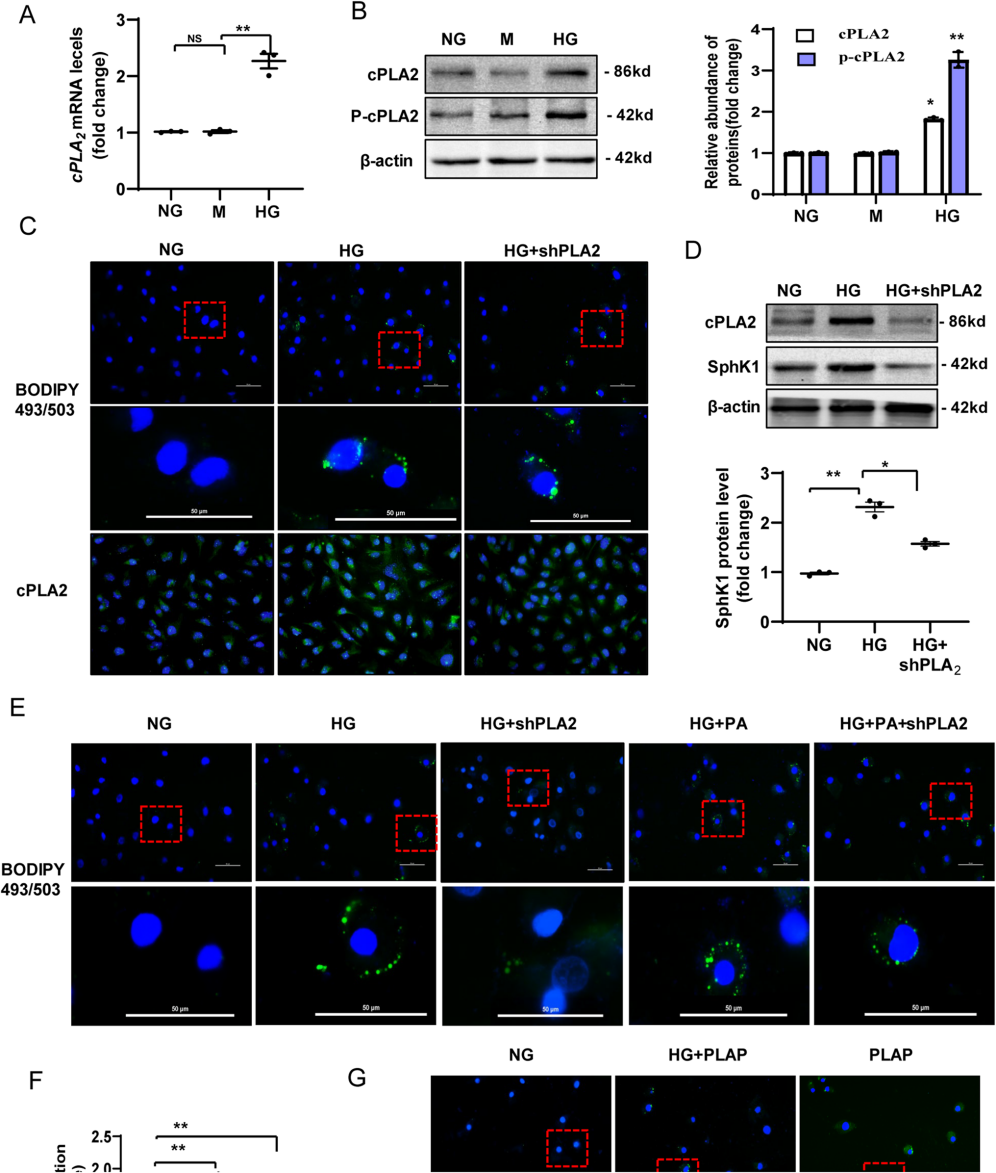
Elevated cPLA2 expression is required for LD formulation and S1P accumulation in HG-induced HK-2 cells. **A** qRT-PCR analysis of cPLA2 mRNA expression in HK-2 cells. **B** Representative western blotting assay and quantitation of the level of cPLA2, and p-cPLA2 in HK-2 cells. **C** Representative images for lipid droplets in HK-2 cells stained with BODIPY 493/503 reagent. **D** Western blotting analysis for the levels of cPLA2 and SPHK1 in shPLA2 infected HK-2 cells. **E** Representative images for lipid droplets in HK-2 cells. **F** The change in the S1P concentration in HK-2 cells in each group after PLAP treatment. **G** Representative images of lipid droplet staining in HK-2 cells of each group after PLAP treatment. NG: 5.6 mM D-glucose; M: NG + 24.4 mM mannitol; HG: 30 mM D-glucose; HG + PA: HG + 300 μM PA; HG + S1P: HG + 1 µM S1P; HG + shPLA2: HG + PLA2 shRNA plasmid, HG + PA + shPLA2: HG + PA + PLA2 shRNA plasmid. PLAP: 1 µM PLAP; HG + PLAP: HG + 1 µM PLAP. Data are expressed as the mean ± SD of three independent experiments. **P < 0.01 versus the NG group; #*P* < 0.05, compared with the HG group using ANOVA

Recent studies demonstrated that cPLA_2_ is critical for the regulation of phospholipid metabolism and LD formation [39]. Therefore, we hypothesized that elevated cPLA_2_ might be involved in the reprogrammed lipid metabolism in diabetic TECs, resulting in lipid peroxidation and cell damage. We first determined the alterations of expression of the gene encoding cPLA_2_ in HG-induced HK-2 cells. We observed an increase in the expression of *CPLA2* in HK-2 cells after HG treatment for 72 h (Fig. 5A). Total cPLA_2_ and phosphorylated cPLA_2_ levels were also elevated in HG-induced HK-2 cells, compared with those in the NG group (Fig. 5B). We next investigated the relationship between cPLA_2_ levels and LD formation in HG-induced TECs using immunofluorescence staining. We observed that cPLA_2_ levels paralleled the accumulation of LDs in HG-induced HK-2 cells, as evaluated using BODIPY 493/503 staining (Fig. 5C). We next determined whether knockdown of *CPLA2* expression using a specific shRNA plasmid could prevent LD formation and S1P accumulation in HG induced-HK-2 cells. Transfection of the *CPLA2*-specific shRNA downregulated cPLA_2_ protein levels (Fig. 5D) and prevented LD formation (Fig. 5E) in HG or HG + PA treated HK-2 cells. Furthermore, knockdown of *CPLA2* expression inhibited the activation of SPHK1 in HK-2 cells in the HG group compared with that in the NG group (Fig. 5D), suggesting downregulation of S1P metabolism. To further identify the role of cPLA2 in the promotion of LD formation and S1P accumulation in HK-2 cells, we also used a gain-of-function strategy using the cPLA2 activator, phospholipase A2-activating protein (PLAP). The results demonstrated that PLAP treatment not only induced S1P accumulation (Fig. 5F) but also promoted LD formation (Fig. 5G), in HK-2 cells with or without high glucose. Collectively, these results suggested that cPLA2 is required for LD formulation and S1P accumulation in HG-induced HK-2 cells.

### HG induction of cPLA2 expression is mtROS dependent

**Fig. 6:**
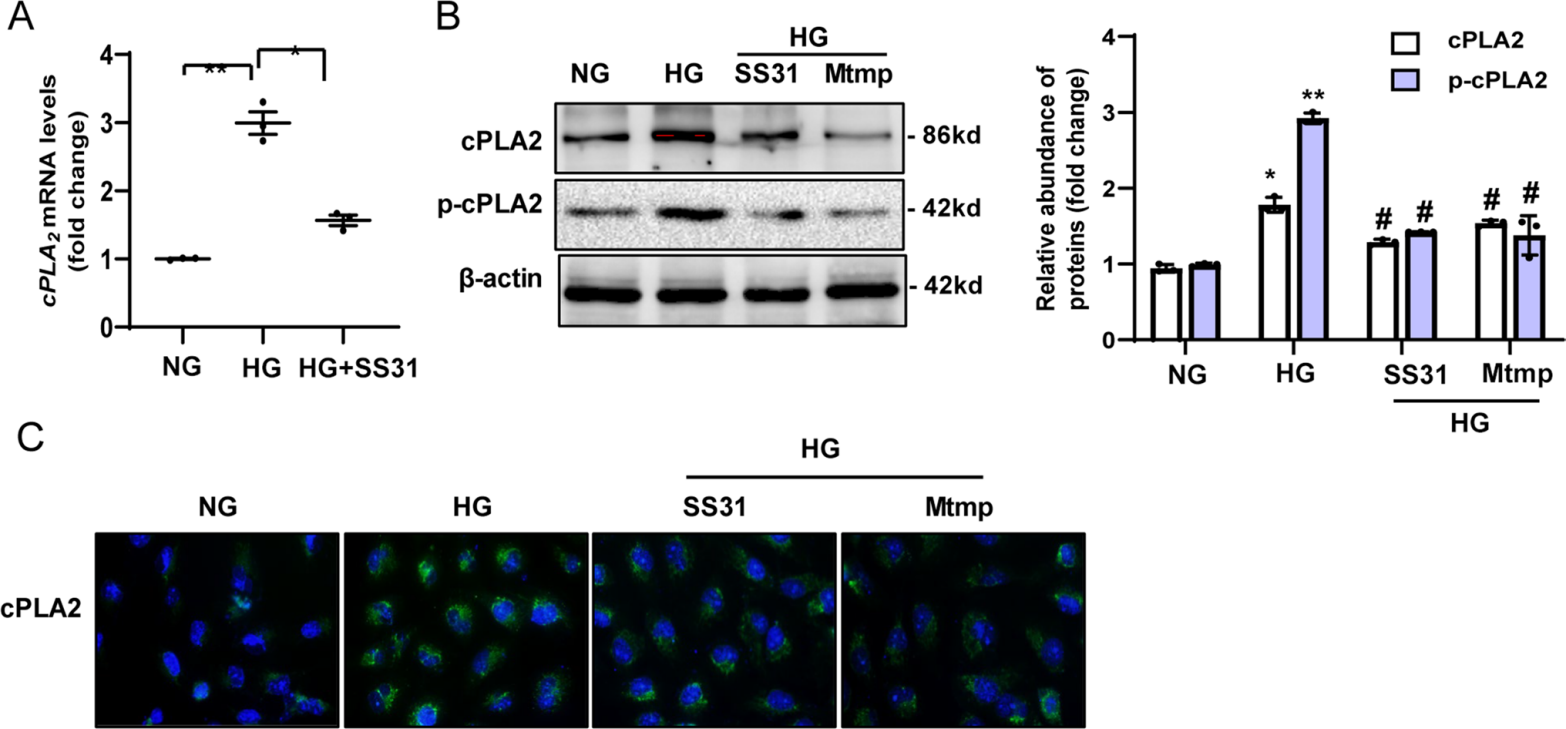
HG induction of cPLA2 expression is mtROS-dependent. **A** qRT-PCR analysis of cPLA2 mRNA expression in HK-2 cells under HG exposure for 72 h, with or without SS31. **B** Western blot analysis for the level of cPLA2 and p-cPLA2 in HK-2 cells under HG exposure for 72 h with SS31 or Mtmp.** C** Representative images for immunofluorescence staining of cPLA2 in HK-2 cells. NG: 5.6 mM D-glucose; HG: 30 mM D-glucose; HG + SS31: HG + 100 nM SS31; HG + Mtmp: HG + 25 μM Mito-Tempo. Data are expressed as the mean ± SD of three independent experiments. **P < 0.01 versus the NG group; ^#^*P* < 0.05, compared with the HG group using ANOVA

Mitochondrial dysfunction has been shown to induce cPLA2 protein synthesis in macrophages [40]; therefore, we determined whether mitochondrial oxidative damage is also related to altered cPLA2 expression in HG-induced HK-2 cells. Improved mitochondrial function in the presence of SS31 markedly increased both the mRNA (Fig. 6A) and protein expression of cPLA2 (Fig. 6B, C) in HK-2 cells after HG treatment for 72 h (Fig. 6A). To evaluate whether mtROS are mechanistically linked with cPLA2 expression under HG conditions, we used a specific mitochondria-targeted antioxidant, MitoTempo, which is a superoxide dismutase mimetic that accumulates in mitochondria. We found that MitoTempo reduced ~ 90% of the increase in cPLA2 expression and phosphorylation in HG induced HK-2 cells (Fig. 6B, C). The results suggested that HG induces cPLA2 expression partly through mtROS.

### Mitochondria-targeted antioxidant inhibits cPLA2f activation in diabetic TECs in vivo

**Fig. 7:**
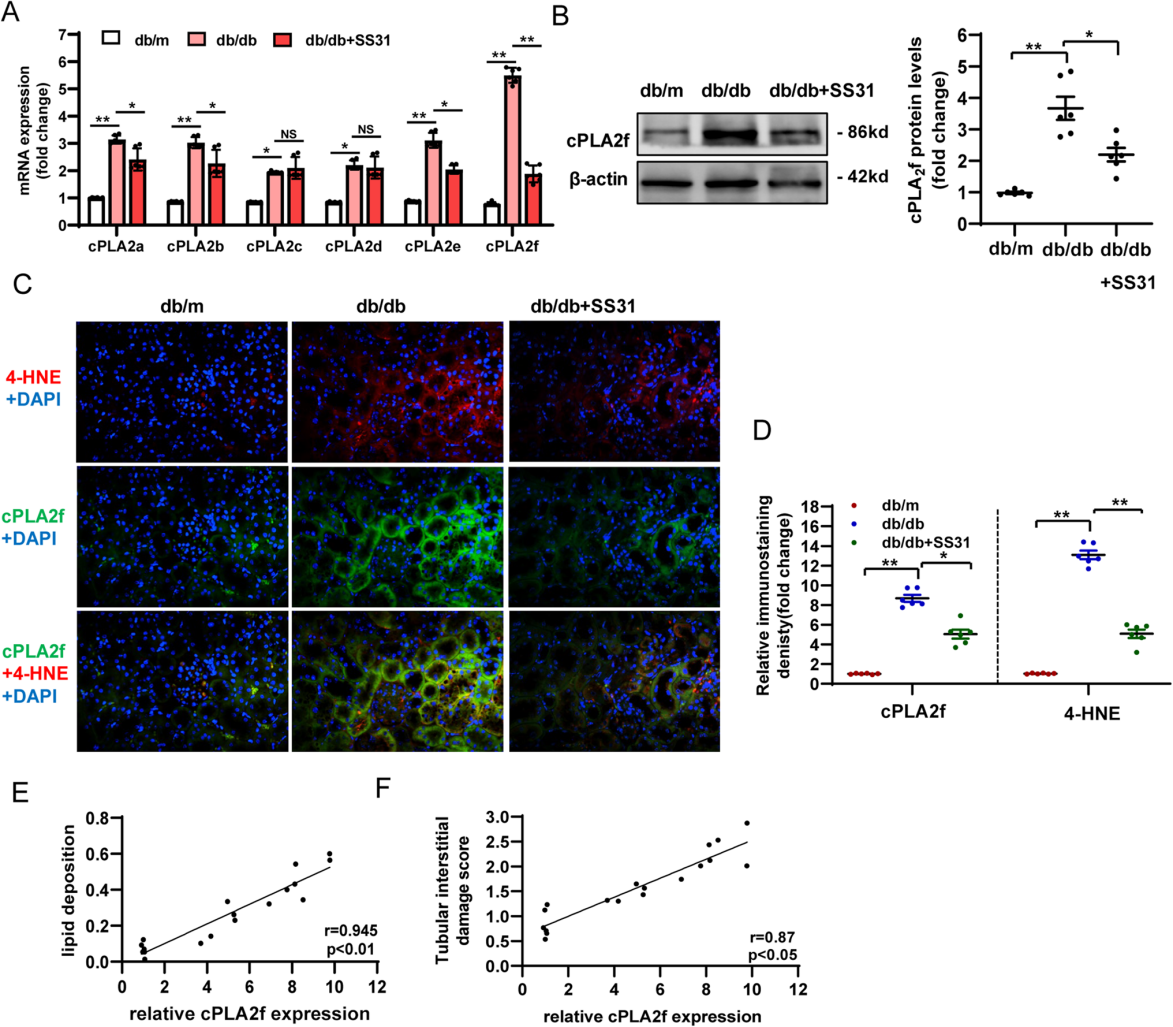
Mitochondria-targeted antioxidant prevents cPLA2f activation in diabetic TECs in vivo.** A** qRT-PCR analysis of cPLA2a, cPLA2b, cPLA2c, cPLA2d, cPLA2e, and cPLA2f mRNA expression in db/m, db/db, and db/db + SS31 mice. **B** Western blotting analysis for the level of cPLA2f in each group mice. **C**, **D** Representative images and quantification for immunofluorescence staining of cPLA2f and 4-HNE in kidney tissues. **E** Correlation analysis of lipid deposition with the level of cPLA2f. (*P* < 0.01, *R* = 0.945). **F** Correlation analysis of lipid deposition with the level of cPLA2f. (*P* < 0.05, *R* = 0.87). db/m: normal male mice; db/db: diabetic mice; db/db + SS31: db/db mice with SS31 treatment; Data are expressed as the means ± SD (*n* = 6). **P < 0.01 versus the db/m group; *P < 0.05, compared with the db/db group using ANOVA

To test the physiological relevance of our in vitro findings, we evaluated the expression of the gene encoding cPLA_2_ in vivo. We first determined the alterations in the level of mRNA encoding isoforms cPLA_2_a–f in each group. Our data showed that the levels of mRNA encoding all cPLA_2_ isoforms were markedly increased in the kidneys of db/db mice, among which the level of the mRNA encoding isoform cPLA2f showed the largest change (Fig. 7A). Observation of the effect of mitochondrial oxidative stress on this change showed a ⁓63% decrease in *Cpla2* mRNA level after SS31 treatment, and the changes cPLA2f mRNA expression and protein level were significantly inhibited by SS31 treatment (Fig. 7B). Immunofluorescent staining and quantitative analysis showed that the level of the lipid peroxidation marker 4-HNE increased in the areas with high expression of isoform cPLA2f (Fig. 7C, D). Positive correlations were observed between the level of isoform cPLA2f and lipid deposition (*r* = 0.945) (Fig. 7C, E), and the isoform cPLA2f level and tubular interstitial damage (*r* = 0.719) in the kidney tissues of db/db mice (Fig. 7C, F). These results suggested that renal expression of isoform cPLA2f, mediated by mitochondrial oxidative stress, is involved in the process of renal diabetic tubular injury.

## Discussion

In this study, we aimed to identify and examine the impact of mitochondrial oxidative damage on metabolic abnormalities under diabetic conditions in TECs. Our results showed that mitochondrial oxidative stress contributes to reprogramming the lipid metabolism of diabetic TECs, including the formation of LDs, S1P accumulation, and impaired FAO and lipid peroxidation. Thus, mitochondrial protection is a potential strategy to treat DKD.

Reprogramming of tubule metabolism is currently considered as a hallmark of kidney disease [4–8]. TECs have a high baseline energy consumption and abundant mitochondria, and thus preferentially use FAO as a source of ATP. Moreover, their low levels of metabolic flexibility make them highly susceptible to metabolic stimuli during diabetes. Recently, study of the metabolic changes of diabetic TECs has introduced the concept of the “Warburg effect”, which was first observed in cancer metabolism [41]. This highlighted that hyperglycemia-derived enhanced glucose entry and glycolysis leads to tubular dysfunction. However, Qi et al. reported that enhanced pyruvate kinase II (PKM2) activity might preserve podocyte function by increasing glucose flux through glycolysis in patients with diabetes, suggesting that enhancing the glucose metabolism pathway has a renal protective effect [42]. A growing body of evidence indicates that sodium-glucose cotransporter 2 inhibitors exert a strong renal protective effect by reducing renal glucose reabsorption in the proximal tubule; however, one of the mechanisms is the protective effects against mitochondrial dysfunction and fatty acid metabolism of TECs [43]. Although the diabetes-induced metabolic switch from oxidative phosphorylation to glycolysis has been observed in diabetic TECs, the metabolic reprogramming related to mitochondrial damage is different from the Warburg effect in tumors, which have normal mitochondrial function. In the present study, we also found that protecting mitochondria and preventing mitochondrial oxidative damage protected against tubular injury and kidney dysfunction by improving dysregulated lipid metabolism. Lipid accumulation has long been considered one of the hallmarks of damaged TECs. The present and previous studies have confirmed the existence of ELD, as shown by increased amounts of LDs and TG [33]. However, research has found that high levels of triglyceride and fatty acids in TECs alone were not sufficient to induce cellular injury, suggesting that mitochondrial damage might play a more important role in the damage caused by lipid deposition [4]. Our results with SS31 provide evidence that mitochondrial dysfunction is the cause of the metabolic changes observed in diabetic TECs, and reducing mitochondrial oxidative stress could reduce LD formation, and improve lipid peroxidation and cellular damage.

Growing evidence suggests that lipotoxicity-associated renal damage depends not only on the quantity of lipids that accumulate in the kidney, but also on the lipid species [6, 44]. In recent years, a clear role of sphingolipids in the pathogenesis of DKD has been established [16]. Sphingolipids, the second largest group of membrane lipids, are the major components of the lipid raft for membrane protein–protein interactions [33]. Among the sphingolipids, the study of S1P and S1P signaling in different renal cells has revealed their contribution to, or association with, the pathogenesis of kidney disease [45]. However, S1P and S1P signaling might differ depending on the renal cell type and could have opposite effects under different kidney diseases [45]. In DKD, recent studies have demonstrated that mice with podocyte-specific deletion of S1P lyase develop proteinuria, and increased glomerular S1P resulting from increased SPHK1 activity is associated with increased mesangial proliferation in STZ-induced diabetes [46, 47]. Exposure to high glucose, S1P and S1PR1/S1PR2-mediated signaling might contribute to fibronectin accumulation in mesangial cells [48]. Although STZ-injected mice showed increased SPHK1 and connective tissue growth factor expression in tubules after 30 days, SPHK1 deficiency further aggravated the DKD fibrosis phenotype, thus the role of sphingolipids in diabetic TECs and the effect of MtROS in this process remain unclear [49]. The results of the present study suggested that mtROS/SPHK1 promote S1P accumulation in diabetic TECs, which is related to lipid peroxidation and cellular damage. Furthermore, we also showed that alterations in the sphingolipid profile in the renal cortex of early-stage DKD, i.e., decreased ceramides and increased S1P, were accompanied by changes in ceramides and key enzymes in metabolic pathways. SS31 intervention had no obvious effect on the de novo synthesis of ceramides, but significantly inhibited the activation of key S1P-related enzymes (an S1P transporter and receptor), which are mainly located in the cell membrane. Our studies connect mitochondrial oxidation stress to the S1P signaling pathway in diabetic TECs. Further studies are required to determine the mechanisms by which mitochondrial damage affects these enzymes and receptors.

Our in vitro study found that both the formation of LDs (neutral lipids) and S1P accumulation were closely related to the activation of cPLA2, which is regulated by mitochondrial oxidation stress. PLA2s are a group of lipolytic enzymes that provide free fatty acids and lysophospholipids from the hydrolysis of the ester bond at the sn-2 position of glycerophospholipids [50]. In recent years, it was suggested that cPLA2 is critical for regulation of phospholipid metabolism and LD formation in various diseases [51]. Early studies demonstrated that in the kidney, activation of cPLA2 represents an important mechanism leading to the development of metabolic alterations that precede cell death during ischemia [24, 26, 52]. Recent observations demonstrated that cPLA2 activity contributed to renal oxidative stress, inflammation, and end-organ damage [22, 23, 26]. These studies also suggested that cPLA2 might be an important mediator of oxidant damage to renal epithelial cells [27]. Our in vivo studies further revealed that cPLA2 is an important enzyme that mediates mitochondrial oxidative signaling and unbalanced lipid metabolism in diabetic TECs.

In addition, another aspect of our study that should be emphasized is that we used a new drug, SS31, as a model for in vitro study. SS-31 has been shown to improve the course of diverse experimental models of kidney diseases associated with mitochondrial oxidative stress [53]. SS-31 is currently in a clinical trial (NCT01755858) to treat acute kidney injury [54]. The mechanism of these effects has generally been ascribed to a reduction in ROS and/or improvement in mitochondrial function and efficiency via the dimethyl-Tyr group [53]. However, recent studies found that the effects of SS31 are thought to be partly mediated by interaction with the inner mitochondrial membrane, especially cardiolipin, the hallmark phospholipid of mitochondria, which plays a crucial role in regulating mitochondrial oxidative stress and might be involved in regulating fatty acid β oxidation [55–57]. Our results also showed that SS31 can help maintain phospholipid homeostasis in DKD via cPLA2. Therefore, it remains possible that restoring the normal phospholipid profile could be a potential strategy for renal protection. Moreover, a previous study found that SS31 can inhibit LD formation in high fat diet-induced kidney disease, which is related to the improvement of mitochondria [58]. Our study also found that LDs induced by type 2 diabetes in the heart, liver, kidney, and muscle tissue were significantly reduced by SS31 treatment (Supplementary Fig. 5A). Interestingly, SS-31 had no effect on organ weight and body weight, but did improve cardiac and renal function in experimental models of diabetes. Improving mitochondrial function is thus believed to be important for DM; however, further research is needed to support such a conclusion. It also suggests that antioxidant treatment, which improves mitochondrial function and promotes lipid consumption, also leads to the accumulation of other metabolites (to maintaining the same organ weight), as shown by the increased synthesis of various amino acids after SS31 intervention in our metabolomic data. Such increased metabolites might also have damaging effects, suggesting that more attention should be paid to metabolite changes in clinically targeted antioxidant therapy.

Although we have shown links between mitochondrial oxidative metabolism and reprogrammed lipid metabolism in diabetic TECs using antioxidants to target mitochondria. However, it is known that diabetes mellitus has the disorder of glucose and lipid metabolism, and oxidative stress also plays an important role in diabetic glomerular cell injury, and the degree of podocyte depletion correlates with disease severity [59]. Recent studies have found that Glomerular endothelial cell (GEC) dysfunction has been attributed to the pathogenesis of DKD [60, 61]. Inhibiting mitochondrial oxidative stress with drugs can delay the progression of DKD by improving the damage of GEC [60]. In fact, we and previous studies have found that SS31 can also improve glomerular podocyte lipid deposition [62]. However, further studies are needed to confirm whether mitochondrial ROS also affect glomerular LD generation and S1P accumulation, whether it is consistent with renal tubule accumulation, or whether there is cell specificity.

In summary, the present study suggests a role for mitochondrial oxidative damage in lipid metabolism-mediated reprogramming of TECs in the diabetic kidney. Mitochondrial protection linked to restoring reprogrammed lipid metabolism is a potential strategy to treat DKD.

### Supplementary Information

Below is the link to the electronic supplementary material.

Supplementary file1 (PDF 318 KB)

Supplementary file2 (PDF 513 KB)

Supplementary file3 (PDF 214 KB)

Supplementary file4 (PDF 3691 KB)

Supplementary file5 (PDF 228 KB)

## Data Availability

Data can be accessed by contacting the corresponding authors.

## Abbreviations

DKD: Diabetic kidney disease
TECs: Tubular epithelial cells
mtROS: Mitochondrial reactive oxygen species
FAO: Fatty acid oxidation
S1P: Sphingosine-1-phosphate
LD: Lipid droplet
cPLA2: Cytosolic phospholipase A2
ELD: Ectopic lipid deposition
SPHK1: Sphingosine kinase 1
HG: High glucose
NG: Normal glucose
M: Mannitol
BG: Blood glucose
KW: Kidney weight
BW: Body weight
Scr: Serum creatinine
TC: Serum total cholesterol
TG: Serum triglycerides
UAE: Urine albumin excretion
PBS: Phosphate-buffered saline
NGAL: Neutrophil gelatinase-associated lipocalin
NAG: N-acetyl-alpha-glucosaminidase
KIM1: Kidney injury molecule 1
OCR: Oxygen consumption rate
LC–MS: Liquid chromatography–mass spectrometry
