# Supplementary material for: Mitochondrial oxidative damage reprograms lipid metabolism of renal tubular epithelial cells in the diabetic kidney
Source: Cell Mol Life Sci. 2024 Jan 11;81(1):23. doi: 10.1007/s00018-023-05078-y (PMC10781825; doi:10.1007/s00018-023-05078-y)
Supplement: Supplementary file 1 — Supplementary file1 (PDF 318 KB) [file 18_2023_5078_MOESM1_ESM.pdf]

Supplementary Figure 1

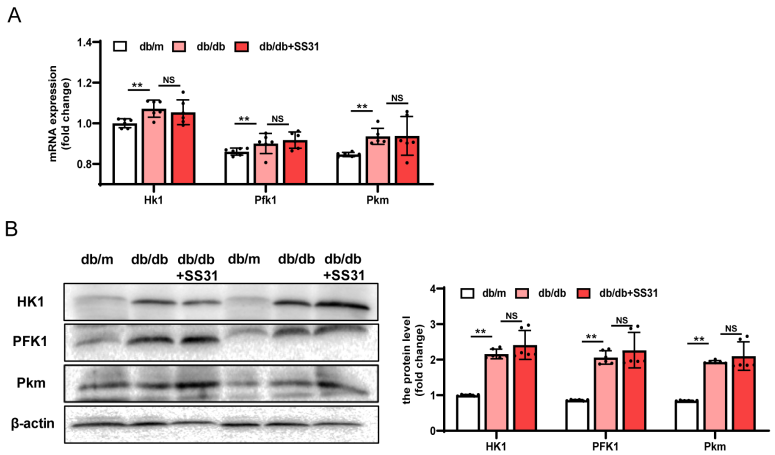

Fig.S 1 **A:** Renal mRNA levels of Hk1, Pfk and Pkm were detected by real-time PCR. **B:** Representative Western blots and semiquantitative analysis of HK1, PFK1 and Pkm. db/m: normal male mice; db/db: diabetic mice; db/db+SS31: db/db mice with SS31 treatment; Data are expressed as means±SD. (n=6). **P<0.01 versus the db/m group; *P<0.05, compared with the db/db group by ANOVA.
