# Supplementary material for: Mitochondrial oxidative damage reprograms lipid metabolism of renal tubular epithelial cells in the diabetic kidney
Source: Cell Mol Life Sci. 2024 Jan 11;81(1):23. doi: 10.1007/s00018-023-05078-y (PMC10781825; doi:10.1007/s00018-023-05078-y)
Supplement: Supplementary file 2 — Supplementary file2 (PDF 513 KB) [file 18_2023_5078_MOESM2_ESM.pdf]

Supplementary Figure 2

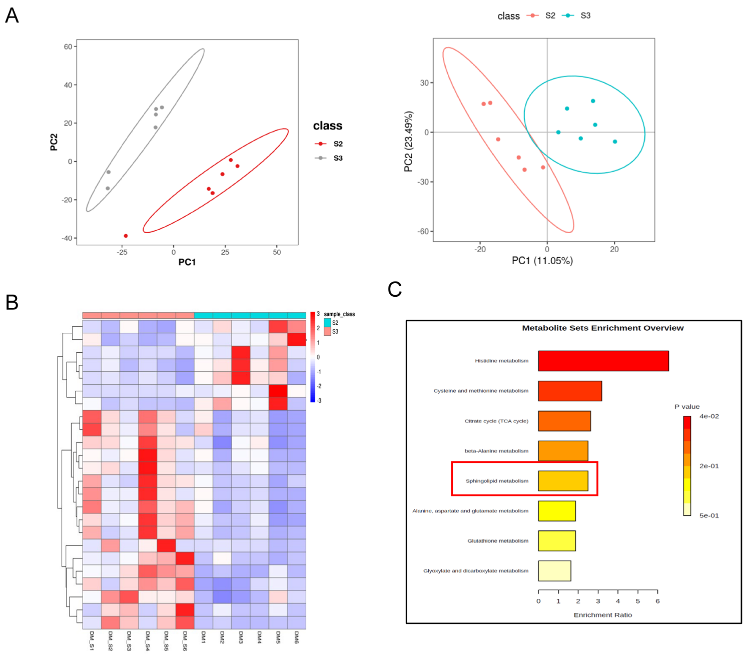

Fig.S 2 A: Orthogonal projections to latent structures discriminant analysis (left: PCA; right: OPLS-DA) plot for comparison of each groups**. B:** Heatmap of differential metabolites between db/db mice diabetic group and db/db + SS31 treatment group. Blue is db/db diabetic group, and red is the db/db + SS31 group. **C:** The disturbed metabolic pathways from comparison between db/db mice diabetic group and db/db + SS31 treatment group. S2: db/db mice diabetic group, S3: db/db + SS31 treatment group.
