# Supplementary material for: Mitochondrial oxidative damage reprograms lipid metabolism of renal tubular epithelial cells in the diabetic kidney
Source: Cell Mol Life Sci. 2024 Jan 11;81(1):23. doi: 10.1007/s00018-023-05078-y (PMC10781825; doi:10.1007/s00018-023-05078-y)
Supplement: Supplementary file 3 — Supplementary file3 (PDF 214 KB) [file 18_2023_5078_MOESM3_ESM.pdf]

Supplementary Figure 3

A

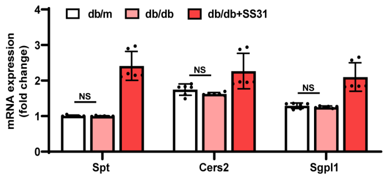

Fig.S 3 A: Renal mRNA levels of Spt, Cers2 and Sgpl1 were detected by real-time PCR. db/m: normal male mice; db/db: diabetic mice; db/db+SS31: db/db mice with SS31 treatment; Data are expressed as means±SD. (n=6). **P<0.01 versus the db/m group; *P<0.05, compared with the db/db group by ANOVA.

Supplementary Figure 4

A

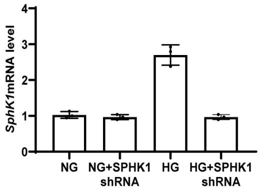

B

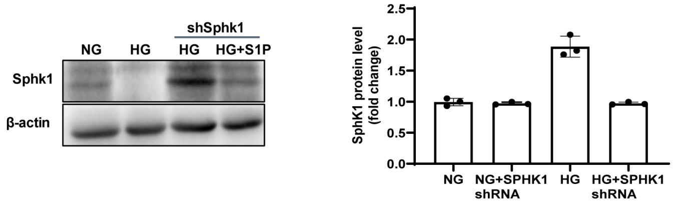

Fig.S 4 A: Renal mRNA levels Sphk1 were detected by real-time PCR. **B:** Representative Western blots and semiquantitative analysis of Sphk1. NG: 5.6 mM D-glucose; HG: 30 mM D-glucose; HG+shSphk1: HG+ Sphk1shRNA plasmid, HG+S1P+shSphk1: HG+ S1P+ Sphk1shRNA plasmid. Data are expressed as mean ± SD of three independent experiments. **P<0.01 versus the NG group; #P<0.05, compared with the HG group by ANOVA.
