# Supplementary material for: Mitochondrial oxidative damage reprograms lipid metabolism of renal tubular epithelial cells in the diabetic kidney
Source: Cell Mol Life Sci. 2024 Jan 11;81(1):23. doi: 10.1007/s00018-023-05078-y (PMC10781825; doi:10.1007/s00018-023-05078-y)
Supplement: Supplementary file 4 — Supplementary file4 (PDF 3691 KB) [file 18_2023_5078_MOESM4_ESM.pdf]

Supplementary Figure 5

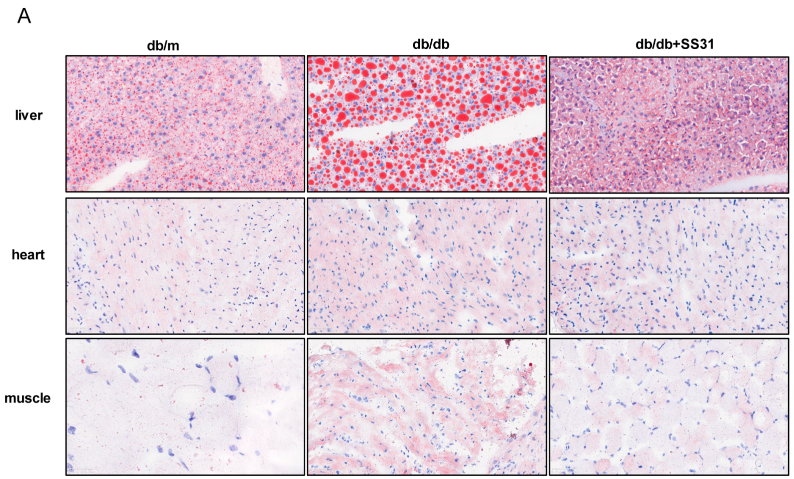

Fig.S 5 A: Representative images for oil red staining. db/m: normal male mice; db/db: diabetic mice; db/db+SS31: db/db mice with SS31 treatment.
