# Supplementary material for: Mitochondrial oxidative damage reprograms lipid metabolism of renal tubular epithelial cells in the diabetic kidney
Source: Cell Mol Life Sci. 2024 Jan 11;81(1):23. doi: 10.1007/s00018-023-05078-y (PMC10781825; doi:10.1007/s00018-023-05078-y)
Supplement: Supplementary file 5 — Supplementary file5 (PDF 228 KB) [file 18_2023_5078_MOESM5_ESM.pdf]

Supplementary Table 1. WB and IF/ ICC antibodies

**Primary antibodies**	**Catalog no.**	**Dilution**	**Supplier**
Rabbit anti-KIM1	ab228973	IF: 1 to 50	Abcam
Mouse anti-4-HNE	ab48506	IF: 1 to 50	Abcam
Rabbit anti-PPARα	bs-23398R	WB: 1 to 1000; IF: 1 to 50	bioss
Rabbit anti-CPT1	15184-1-AP	WB: 1 to 1000	Proteintech
Rabbit anti-ACOX1	AB184032	WB: 1 to 1000;	Abcam
Rabbit anti-PGC-1	SAB2108742	WB: 1 to 1000; IF: 1 to 50	Sigma
Mouse anti-AMPKa	GTX60403	WB: 1 to 1000	GeneTex
Rabbit anti-pAMPKa	2535	WB: 1 to 1000	CST
Rabbit anti-CDase	DF9192	WB: 1 to 1000	Affinty
Rabbit anti-Sphk1	10670-1-AP	WB: 1 to 1000; IF: 1 to 50	Proteintech
Rabbit anti-Spns2	AB82629	WB: 1 to 1000	Abcam
Rabbit anti-S1pr1	AB11424	WB: 1 to 1000	Abcam
Mouse anti-α-SMA	BM0002	WB: 1 to 1000	Bosterbio
Rabbit anti-E-ca	3195T	WB: 1 to 1000	CST
Rabbit anti-cPLA2	5479	WB: 1 to 1000; ICC: 1 to 50	CST
Rabbit anti- P-cPLA2	53044	WB: 1 to 1000	CST
Rabbit anti-cPLA2f	DF9430	WB: 1 to 1000; IF: 1 to 50	Affinty
Mouse anti-β-actin	BM0627	WB: 1 to 1000	Bosterbio

Supplementary Table 2. Sequences of the realtime PCR Primers

Genes	Forward Primers (5'-3')	Reverse Primers (5'-3')
Cd36	CACAGCTGCCTTCTGAAATGTGTGG	TTTCTACGTGGCCCGGTTCTAATTC
Fabp4	GGCATGGCCAAACCTAACAT	TGTACCAGGACACCCCCATC
Srebf1	GTGAGGCGGCTCTGGAACAGAC	ATAGGGGGCGTCAAACAGGCC
Fasn	GGCTCTATGGATTACCCAAGC	CCAGTGTTCGTTCCTCGGA
Acaca	CAACACTGGGCGCAGGTTTA	CAACACTGGGCGCAGGTTTA
Scd1	ACCATCACAGCACCTCCTTC	ATTTCAGGGCGGATGTCTTC
Pparα	CAATGGAGATGGTGGACACA	TTGTAGGAAGTCTGCCGAGAG
cpt1a	GATCTACAATTCCCCTCTGCTCT	TAGAGCCAGACCTTGAAGTAACG
acox1	TATTCGGCTATGACTGGGCACA	GATG-GATACTTTCTCGGCAGGA
Hk1	AAAGCGAGGGGACTATGA	ATCAATGTGCCTCAGTTCC
Phk1	TGTGGTCCGAGTTGGTATCTT	GCACTTCCAATCACTGTGCC
Pkm	GTGGCTCGGCTGAATTTCTCT	GTGGCTCGGCTGAATTTCTCT
Acer1	TCTGAGGTGGATTGGTGTGAG	TGAGGGGTCCAAAGATGAGGA
Spt	TCCTCTTGATCGCTTCCAAAGG	GGCTTTTTGTCTAAGCTCCTCTT
Cers2	ATGCTCCAGACCTTGTATGACT	CTGAGGCTTTGGCATAGACAC
Sgpl1	CTGAAGGACTTCGAGCCTTATTT	CTGAAGGACTTCGAGCCTTATTT
SphK1 *	GCTCTGGTGGTCATGTCTGG	GCTCTGGTGGTCATGTCTGG
cPla2 *	GGCGGGAAACCTACCCATAC	CCACAGTTGCACATAGCAGT
cPla2a	CAGCACATTATAGTGGAACACCA	AGTGTCCAGCATATCGCCAAA
cPla2b	TGGCCCCTAGCCAACTTTG	TGGCCCCTAGCCAACTTTG
cPla2c	AGGAGCTGAAACATCGGTATGA	CTGCAAAGATGGGATAGGGC
cPla2d	CCATCCTTACCAGGAGGAGG	TGGAGTTAGTGACTGTTTGGGT
cPla2e	ATGGTGACAGACTCCTTCGAG	CCTCTGCGTAAAGCTGTGG
cPla2f	AGCCATACTGCTACGGAAGAC	TTTGGACAACTTATCTGTGTGCT
18s	ACACGGA CAGGATTGACAGA	GGACATCTAAGGGCATCACAG

*Human

Supplementary Table 3.

ID	Metabolite	ratio	t.test_p.value	t.test_p.value_BHcorrec	VIP	regulated
pos-0.964_260.05078	suprofen	1.345029176	0.049058836	0.999216615	1.778286	up
pos-1.572_189.06339	aminodhq	1.91883867	0.046595085	0.999216615	2.182525	up
pos-3.011_87.10479	isoamylamine	2.403145466	0.046579771	0.999216615	0.965273	up
neg-4.321_225.13639	terbutaline	1.251426113	0.042040581	0.995485198	1.790896	up
pos-3.734_232.08443	nalidixic acid	1.456722866	0.041888044	0.999216615	1.923989	up
pos-0.772_240.02344	l-cystine	1.302309068	0.041806489	0.999216615	1.432361	up
neg-3.753_232.0846	nalidixic acid	1.500196343	0.03531295	0.995485198	2.242636	up
neg-1.366_192.0268	citric acid	5.057683575	0.03226811	0.995485198	2.08456	up
neg-0.903_112.0159	2-furoate	3.02321469	0.030022557	0.995485198	2.283844	up
pos-2.715_159.0683	3-methyl-quinolin-2-ol	1.693582582	0.027617335	0.999216615	1.704483	up
pos-3.738_129.05781	quinoline	1.486236588	0.027546471	0.999216615	2.180134	up
pos-0.883_244.06909	pseudouridine	1.214621851	0.026848482	0.999216615	1.881152	up
pos-9.804_311.12588	imazaquin	1.426737931	0.025872051	0.999216615	1.88121	up
neg-3.126_86.03667	crotonic acid	41.89435072	0.025000503	0.995485198	2.058501	up
neg-3.291_177.04577	n-formyl-l-methionine	1.881885885	0.023666756	0.995485198	1.446308	up
pos-3.318_205.0376	xanthurenic acid	1.569062009	0.019532163	0.999216615	1.022362	up
neg-3.418_145.05264	1(2h)-isoquinolinone	1.611893372	0.018585595	0.995485198	2.473893	up
neg-0.742_226.10647	carnosine	1.662403392	0.016805154	0.995485198	1.419471	up
neg-0.84_158.04364	(r)(-)-allantoin	1.583720325	0.01556653	0.995485198	1.907263	up
pos-3.638_285.10329	probenecid	2.620980812	0.013941072	0.999216615	2.218814	up
pos-3.521_146.03668	coumarin	2.070601659	0.047058777	0.999216615	1.386331	up
pos-1.342_188.11575	6-acetamido-3-aminohexanoate	1.68860826	0.046444273	0.999216615	2.285881	up
pos-0.881_254.12615	midodrine	1.491980867	0.046088752	0.999216615	1.418811	up
pos-3.739_304.10289	vicine	2.450435261	0.045964138	0.999216615	2.092452	up
pos-3.308_297.08976	5'-s-methyl-5'-thioadenosine	1.366392772	0.039538598	0.999216615	2.211922	up
neg-3.837_342.13147	coniferin	3.876808225	0.033483079	0.995485198	2.13873	up
neg-3.337_205.03737	xanthurenic acid	1.705330387	0.030321183	0.995485198	1.221983	up
neg-0.902_174.01618	trans-aconitic acid	1.445849225	0.03008955	0.995485198	2.709393	up
neg-3.517_176.06826	2-isopropylmalic acid	1.676147123	0.028732288	0.995485198	1.6486	up
pos-3.474_180.05345	nicotinuric acid	2.932233781	0.021031368	0.999216615	2.809489	up
pos-1.38_250.06201	gamma-glutamylcysteine	1.453525102	0.018158003	0.999216615	2.181422	up
pos-0.885_216.07431	8-hydroxyalanylclavam	1.355554475	0.016459101	0.999216615	2.493403	up
pos-3.403_196.12128	fasoracetam	1.905480492	0.014942857	0.999216615	2.563992	up
pos-4.728_299.28183	d-sphingosine	1.989727494	0.014811645	0.999216615	2.652118	up
pos-4.567_285.26612	c17 sphingosine	2.195959587	0.013682936	0.995485198	1.530609	up
neg-3.474_444.1746	difenacoum	2.736027548	0.011254393	0.995485198	2.951155	up
neg-0.842_156.05338	4-imidazolone-5-propanoate	2.02366249	0.010805049	0.995485198	3.411759	up
pos-0.809_165.04566	l-methionine sulfoxide	1.667202354	0.007146735	0.999216615	1.678909	up
pos-3.343_269.10829	fenothiocarb sulfoxide	4.277163592	0.003110912	0.952872474	2.639896	up
pos-3.388_285.10348	probenecid	6.211689667	0.001420755	0.621681935	3.283284	up
pos-4.756_224.14073	(+/-)-6-hydroxy-3-oxo-alpha-io	0.852675962	0.049548706	0.999216615	1.315322	down
pos-3.233_503.27477	mycalamide a	0.705828732	0.049450927	0.999216615	1.461809	down
pos-3.447_300.11205	desmedipham	0.719960085	0.045508164	0.999216615	1.209003	down
pos-6.149_120.05751	phenylacetaldehyde	0.199159987	0.043835625	0.999216615	1.875731	down
neg-3.516_217.1311	o-propanoylcarnitine	0.720445684	0.042265456	0.995485198	1.12677	down
pos-0.813_117.07879	betaine	0.802382752	0.041786837	0.999216615	2.022711	down
pos-0.802_254.05674	5-l-glutamyl-taurine	0.723645591	0.037904748	0.999216615	1.820923	down
pos-0.888_211.0967	zalcitabine	0.2490287	0.037027003	0.999216615	2.111219	down
neg-3.124_203.07925	n2-acetyl-l-aminoadipate	0.595685257	0.033555506	0.995485198	0.695216	down
neg-3.333_172.01933	p-toluenesulfonic acid	0.080793444	0.033076764	0.995485198	2.152947	down
neg-1.408_337.06755	5-hydroxymethyldeoxycytidylate	0.397303249	0.032063258	0.995485198	2.491772	down
pos-1.613_179.09449	phenacetin	0.409167333	0.030003539	0.999216615	2.258032	down
pos-4.166_164.08348	4-phenylbutyric acid	0.511272771	0.029789437	0.999216615	1.232304	down
neg-0.915_177.94297	pyrophosphate	0.823058526	0.026651953	0.995485198	1.570991	down
neg-0.796_254.05714	5-l-glutamyl-taurine	0.704028798	0.015733276	0.995485198	2.473922	down
neg-4.137_139.02677	6-hydroxynicotinic acid	0.827518136	0.013967259	0.995485198	1.875475	down
pos-4.074_515.29088	taurocholate	0.341261451	0.010069186	0.999216615	1.43204	down
pos-3.547_475.29891	netilmicin	0.714908156	0.006551238	0.999216615	1.25114	down
pos-0.881_167.05794	3-hydroxy-4-methylanthranilate	0.780188901	0.002627847	0.999216615	1.677679	down
neg-4.088_515.29168	taurocholate	0.321610207	0.001760639	0.995485198	1.672785	down
neg-0.809_246.05043	glycerophosphoglycerol	0.723324609	0.000109267	0.995485198	1.892328	down
pos-4.104_179.0943	phenacetin	0.233687877	0.049257162	0.999216615	2.558988	down
neg-0.801_74.03647	(r)-lactaldehyde	0.599765984	0.047986016	0.995485198	2.467496	down
neg-1.501_384.1216	s-adenosylhomocysteine	0.777253731	0.043945565	0.995485198	1.886084	down
pos-0.922_111.99242	hydroxymethylphosphonate	0.611346681	0.041207833	0.999216615	1.971634	down
pos-3.188_75.03197	glycine	0.502861701	0.030307685	0.999216615	1.284167	down
neg-0.923_111.99234	hydroxymethylphosphonate	0.696133726	0.019528007	0.995485198	2.982042	down
neg-3.174_75.03191	glycine	0.529215619	0.01844057	0.995485198	1.36575	down
pos-3.688_333.16069	ipconazole	0.646346876	0.015218876	0.999216615	2.831941	down
neg-3.185_146.0578	(r)-4-dehydropantoate	0.705700024	0.009987056	0.995485198	1.300061	down
pos-0.849_125.01449	taurine	0.742653027	0.008914399	0.999216615	1.688694	down
